# Comparison of Different Surgical Methods for Necrotizing Pancreatitis: A Meta-Analysis

**DOI:** 10.3389/fsurg.2021.723605

**Published:** 2021-09-22

**Authors:** Jianguo Xiao, Xiaojiao Quan, Fang Liu, Wen Li

**Affiliations:** ^1^Medicinal School of Chinese People's Liberation Army, Beijing, China; ^2^Department of Critical Care Medicine, The First Medical Center, Chinese People's Liberation Army General Hospital, Beijing, China; ^3^Department of Critical Care Medicine, Hainan Hospital of Chinese People's Liberation Army General Hospital, Sanya, China; ^4^Department of Gastroenterology and Hepatology, The First Medical Center, Chinese People's Liberation Army General Hospital, Beijing, China

**Keywords:** necrotizing pancreatitis, minimally invasive surgery, endoscopic step-up approach, meta-analysis, open necrosectomy

## Abstract

**Purpose:** To compare the effectiveness and safety of three methods of open necrosectomy, minimally invasive surgery and endoscopic step-up approach for necrotizing pancreatitis.

**Methods:** We searched Pubmed, Embase, ScienceDirect, and CNKI full text database (CNKI) (to December 25, 2019). RCT, prospective cohort study (PCS), and retrospective cohort study (RCS) comparing the effectiveness and safety of any two of above-mentioned three methods were included.

**Results:** There was no significant difference in major complications or death, and mortality between the minimally invasive surgery treatment group and the endoscopic step-up approach treatment group (RR = 1.66, 95%CI: 0.83–3.33, *P* = 0.15; RR = 1.05, 95%CI: 0.59–1.86, *P* = 0.87); the incidence rate of new-onset multiple organ failure, enterocutaneous fistula, pancreatic-cutaneous fistula, intra-abdominal bleeding, and endocrine pancreatic insufficiency in the endoscopic step-up approach treatment group was significantly lower than minimally invasive surgery group (RR = 2.65, 95%CI: 1.10–6.36, *P* = 0.03; RR = 6.63, 95%CI: 1.59–27.60, *P* = 0.009; RR = 7.73, 95%CI: 3.00–19.89, *P* < 0.0001; RR = 1.91, 95%CI: 1.13–3.24, *P* = 0.02; RR = 1.83, 95%CI: 1.9–3.16, *P* = 0.02); hospital stay in the endoscopic step-up approach group was significantly shorter than minimally invasive surgical treatment group (MD = 11.26, 95%CI: 5.46–17.05, *P* = 0.0001). The incidence of pancreatic-cutaneous fistula in the endoscopic escalation step therapy group was significantly lower than that in the open necrosectomy group (RR = 0.11, 95%CI: 0.02–0.58, *P* = 0.009).

**Conclusion:** Compared with minimally invasive surgery and open necrosectomy, although endoscopic step-up approach cannot reduce the main complications or death and mortality of patients, it can significantly reduce the incidence of some serious complications, such as pancreatic-cutaneous fistula, enterocutaneous fistula, intra-abdominal bleeding, endocrine pancreatic insufficiency, and can significantly shorten the patient's hospital stay.

## Introduction

Acute pancreatitis is a common gastrointestinal disease with a lethal risk. It is divided into edema pancreatitis and necrotic pancreatitis. Necrotic pancreatitis accounts for about 20% of acute pancreatitis (1). Most of the necrotic pancreas or tissues around the pancreas are still sterile, but 30% will continue infection (2, 3). Due to the higher morbidity and mortality (8–39%) of necrotizing pancreatitis (4), it is particularly important to take active and effective treatment measures. The treatment of necrotic pancreatitis includes basic treatment, conservative medical treatment and surgical treatment. The traditional surgical method is open necrosectomy, usually through a bilateral incision under the costal margin or a median incision to completely remove the necrotic tissue. With the continuous development of surgical operations, minimally invasive surgery and endoscopic step-up approach have gradually replaced open necrosectomy. Several studies have compared the effectiveness and safety of different surgical procedures. In view of the inconsistency of the conclusions of these studies, this article uses a systematic review and meta-analysis to compare open necrosectomy, minimally invasive surgery, and endoscopic step-up approach regarding the effectiveness and safety.

## Materials and Methods

### Search Strategy

A search of Pubmed, Embase, ScienceDirect, and CNKI full text database (CNKI) was performed (form establishment of the database to December 25, 2019). Keywords are “necrotizing pancreatitis,” “acute pancreatitis,” “infected necrotizing pancreatitis,” “infected necrosis,” “open necrosectomy,” “endoscopic step-up approach,” “minimally invasive surgery,” “endoscopy,” “endoscopic transgastric drainage,” “endoscopic transgastric necrosectomy,” “percutaneous catheter drainage,” “video-assisted retroperitoneal debridement,” “laparoscopic debridement,” “ETD,” “ETN,” “PCD,” and “VARD.” In order to prevent omissions, references of included studies were also screened. Publication language is Chinese or English. All records were imported into endnote X9 (Thomson Reuters, New York, USA), duplicate documents were removed, and then the titles, abstracts and full texts were screened to obtain studies that meet the entry criteria.

### Inclusion and Exclusion Criteria

Inclusion criteria: (1) randomized controlled trial (RCT), prospective cohort study (PCS) or retrospective cohort study (RCS); (2) Patients with acute necrotizing pancreatitis (whether or not infected); (3) Interventions: open necrosectomy, percutaneous catheter drainage (PCD), video-assisted retroperitoneal debridement (VARD), laparoscopic debridement, endoscopic transgastric drainage (ETD), endoscopic transgastric necrosectomy (ETN); (4) Endpoint outcomes: major complications or death, mortality, new-onset multiple organ failure, enterocutaneous fistula, pancreatic-cutaneous fistula, intra-abdominal bleeding, length of hospital stay, length of ICU stay, endocrine pancreatic insufficiency, and exocrine pancreatic insufficiency.

### Quality Assessment

Two researchers assessed the included literature according to the Cochrane Handbook 5.1.0 quality evaluation standard. The quality evaluation criteria include the following 7 aspects: (1) random sequence generation; (2) allocation concealment; (3) blinding of participants and personnel; (4) blinding of outcome assessment; (5) incomplete outcome data; (6) selective reporting; (7) other bias. Each item is divided into 3 levels: low bias risk, unclear, and high bias risk.

### Data Extraction

Data extracted includes the time of publication, name of the first author, type of study, country, sample size, intervention and control measures, follow-up time, patient characteristics (age, gender, body mass index, disease cause, duration of symptoms, disease severity, Surgery type, infection necrosis ratio, etc.), inclusion criteria, exclusion criteria, endpoint outcomes, etc.

The literature search, literature evaluation and data extraction were performed by two researchers independently. A third reviewer would be invited if there were any disagreements.

### Statistical Analysis

Continuous variables are described as mean difference (MD) and 95% confidence intervals (95% confidence intervals, 95% CIs), and binary variables are described as relative risk (RR) and 95% Confidence interval (95% CIs). *P* < 0.05 indicates that the difference is statistically significant. For statistical analysis, Revman software (version 5.3; Cochrane Collaboration, Copenhagen, Denmark) was used. First, the Cochran's Q statistical test and *I*^2^-test were used to evaluate the heterogeneity between the studies. If *I*^2^ ≥ 50% or *P* < 0.05, then there was a large heterogeneity between the studies. A random effect model was used and a sensitivity analysis was conducted; If *I*^2^ < 50% or *P* > 0.05, it is considered that there is no heterogeneity or the heterogeneity is small, and a fixed effect model is used.

## Results

### Literature Screening and Characteristics of Included Studies

**Figure 1:**
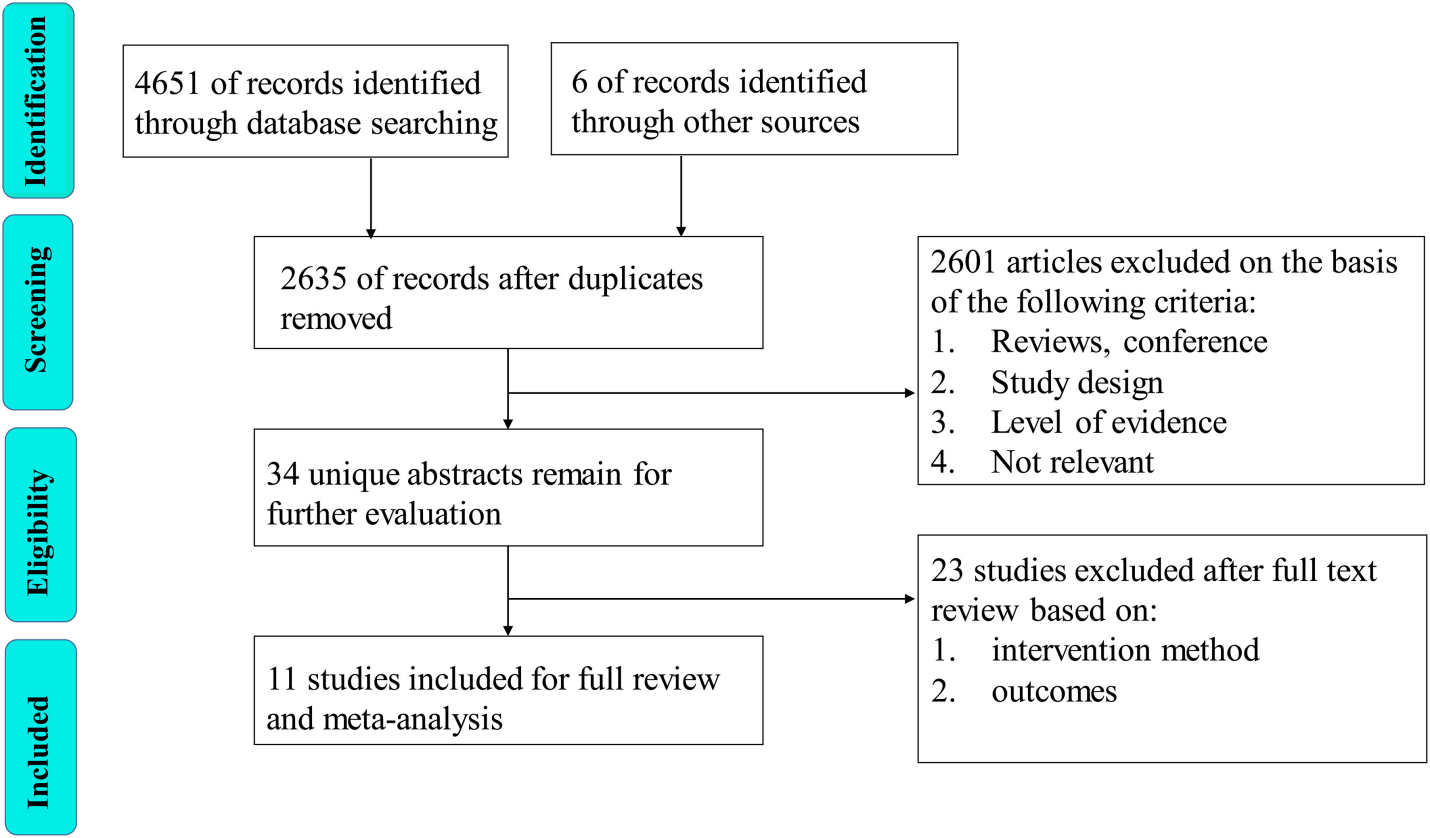
The literature selection process.

A total of 4,657 articles including 4,651 initially retrieved and 6 references found by searching the references of the included articles were identified. Two thousand twenty-two duplicate articles were eliminated by EndNote software. Two thousand six hundred one articles were excluded by reading the title and abstract. Twenty-three articles were removed by reading the full text, and finally 11 articles were identified as eligible. The literature selection process is shown in Figure 1. All 11 articles included 5 RCT (5–9), 2 PCS (10, 11), and 4 RCS (12–15). Eight articles compared the effectiveness and safety of minimally invasive surgery and endoscopic step-up approach, 4 articles compared the effectiveness and safety of minimally invasive surgery and open necrosectomy, and 3 articles compared the effectiveness and safety of endoscopic step-up approach and open necrosectomy (Table 1). The characteristics of baseline pancreatitis and the severity of the disease are shown in Table 2.

**Table 1:** Baseline characteristics of included studies.

**References**	**Study period**	**Country**	**Study design**	**Center**	**Intervention**	**Number of patients**	**Age (years)**	**Male sex: *n* (%)**	**BMI**	**Follow (months)**
Bang et al. (5)	2014.5–2017.3	USA	RCT	Single center	VARD or LD/ ETD+ETN	32/34	52.9 (14.2)/ 55.6 (14.2)	21 (65.6)/ 22 (64.7)		6
van Brunschot et al. (6)	2011.9–2015.1	Netherlands	RCT	multicenter	PCD+VARD/ ETD+ETN	47/51	60 (11)/ 63 (14)	29 (62)/ 34 (67)	28 (25–30)/ 29 (25–32)	6
Bakker et al. (7)	2008.8–2010.3	Netherlands	RCT	multicenter	VARD/ ETN	10/10	64 (46–72)/ 62 (58–70)	8 (80)/ 6 (60)	27 (23–37)/ 29 (26–35)	6
Litvin and Khokha (16)	2004.1–2008.12	Republic of Belarus	RCT	multicenter	PCD+VARD/ ON	37/35				
Van Santvoort et al. (8)	2005.10–2008.11	Netherlands	RCT	multicenter	PCD+VARD/ ON	43/45	57.6 (2.1)/ 57.4 (2.0)	31 (72)/ 33 (73)	28 (20–55)/ 27 (22–39)	3–6
He et al. (10)	2013.5–2014.12	China	PCS	Single center	PCD+VARD/ ETD+ETN	13/13	48 (43–59)/ 48 (27–55)	7 (53.8)/ 5 (45.5)	23 (22–24)/ 23 (22–24)	12
Kumar et al. (11)	2009.1–2010.12	USA	PCS	Single center	PCD+VARD/ ETN	12/12	53.3 (3.0)/ 58.9 (3.9)	9 (75)/ 8 (66.7)	29.5 (2.2)/ 27.0 (1.4)	30 (9.6)/ 22.8 (3.6)
Khreiss et al. (12)	2008–2013	USA	RCS	Single center	VARD or LD/ ETD+ETN	20/20	55 (37–60.5)/ 55 (42.5–66)	16 (80)/ 9 (45)	30.1 (7.4)/ 29.8 (7.3)	6 (3–10)/ 16 (7–24)
Woo et al. (13)	2011.1–2016.12	Australian	RCS	Single center	PCD+VARD/ ETN/ON	8/12/10	60 (32–72)/ 69 (31–81)/ 56 (37–77)	6 (75)/ 8 (67)/ 10 (100)		
Tan et al. (14)	2011.5–2011.9	France	RCS	multicenter	ETN/ ON	11/21	51 (42–57)/ 52 (47–60)	9 (82)/ 14 (67)		16 (median)
Bausch et al. (15)	2002–2010	Germany	RCS	Single center	PCD+VARD/ ETD+ETN/ ON	14/18/30	61 (20–5)/ 58 (15–84)/ 64 (25–88)	11 (79)/ 10 (56)/ 17 (57)		

*RCT, randomized controlled trial; PCS, prospective cohort study; RCS, retrospective cohort study; VARD, video-assisted retroperitoneal debridement; PCD, percutaneous catheter drainage; LD, laparoscopic debridement; ETD, endoscopic transgastric drainage; ETN, endoscopic transgastric necrosectomy; ON, open necrosectomy*.

**Table 2:** Baseline characteristics of the pancreatitis and disease severity.

**References**	**Cause of pancreatitis,** ***n* (%)**	**Intervention**	**Infected necrosis *n* (%)**	**Days of symptoms**	**ASA class III/IV: *n* (%)**	**APACHE II score**	**Disease severity:** ***n*** **(%)**			
							**SIRS**	**ICU admission**	**Single organ failure**	**Multiple organ failure**
Bang et al. (5)	Biliary: 8 (25.0)/14 (41.2) Alcohol: 11 (34.4)/6 (17.6) Idiopathic: 11 (34.4)/14 (41.2) Hypertriglyceridemia: 1 (3.2)/0 (0) Medication: 1 (3.2)/0	VARD or LD/ ETD+ETN	30 (93.8)/31 (91.2)	<28 d: 7 (22)/9 (27) 28–42 d: 16 (50)/19 (56) >42 d: 9 (28)/6 (18)	31 (96.9)/ 32 (94.1)	27.1 (20.3)/ 33.7 (13.5)	16 (50.0)/ 16 (47.1)	4 (40)/ 5 (50)	3 (9.4)/ 2 (5.9)	7 (21.9)/ 7 (20.6)
van Brunschot et al. (6)	Biliary: 30 (64)/26 (51) Alcohol: 7 (15)/7 (14) Other: 10 (21)/18 (35)	PCD+VARD/ ETD+ETN	46 (98%)/ 46 (90%)	41 (28–52)/ 39 (28–54)	2 (4)/ 5 (10)	10 (6–13)/ 9 (5–13)	5 (10)/ 33 (65)	25 (53)/ 21 (41)	14 (30)/ 13 (25)	7 (15)/ 9 (18)
Bakker et al. (7)	Biliary: 7 (70)/6 (60) Alcohol: 2 (20)/2 (20) Other: 1 (10)/2 (20)	VARD/ ETN	9 (90)/ 10 (100)	59 (29–69)/ 48 (36–74)	1 (10)/ 0 (0)	11 (7–14)/ 10 (6–14)	7 (70)/ 9 (90)	3 (30)/ 2 (20)	3 (30)/ 2 (20)	1 (10)/ 2 (20)
Litvin and Khokha (16)		PCD+VARD/ ON								
Van Santvoort et al. (8)	Biliary: 26 (60)/29 (64) Alcohol: 3 (7)/5 (11) Other: 14 (33)/11 (24)	PCD+VARD/ ON	39 (91)/ 42 (93)	30 (11–71)/ 29 (12–155)	13 (30)/ 14 (31)	14.6 (6.1)/ 15.0 (5.3)	42 (98)/ 45 (100)	28 (65)/ 29 (64)	21 (49)/ 22 (49)	15 (35)/ 13 (29)
He et al. (10)	Biliary: 7 (53.8)/5 (45.5) Alcohol: 2 (15.4)/4 (36.4) Hypertriglyceridemia: 4 (30.8)/1 (9.1) Hypercalcemia: 0 (0)/1 (9.1)	PCD+VARD/ ETD+ETN		30 (25–36)/ 27 (22–41)		10 (8–14)/ 7 (6–10)		11 (84.6)/ 8 (72.7)	8 (61.5)/ 7 (63.6)	5 (38.5)/ 1 (9.1)
Kumar et al. (11)	Biliary: 5 (42)/7 (58) Alcohol: 3 (25)/3 (25) Hypertriglyceridemia:1 (8.3)/0 (0) Other:3 (25)/2 (16.7)	PCD+VARD/ ETN				9.4 (1.2)/ 10.1 (1.1)			1 (8.3)/ 0 (0)	0 (0)/ 0 (0)
Khreiss et al. (12)	Biliary: 13 (65)/9 (45) Alcohol: 3 (15)/3 (12) Idiopathic: 3 (15)/2 (10) Other: 1 (5)/6 (30)	VARD or LD/ ETD+ETN								
Woo et al. (13)	Biliary: 2 (25)/8 (67)/5 (50) Alcohol: 1 (12.5)/0 (0)/2 (20) Post-ERCP: 1 (12.5)/1 (8)/1 (10) Other: 4 (50)/3 (25)/2 (20)	PCD+VARD/ ETN/ON								
Tan et al. (14)	Biliary: 5 (45)/6 (29) Alcohol: 4 (36)/6 (29) Other: 2 (18)/9 (43)	ETN/ ON	10 (91)/ 19 (90)	22 (9–74)/ 21 (3–120)						
Bausch et al. (15)	Biliary: 4 (29)/5 (28)/4 (13) Alcohol: 3 (21)/4 (22)/5 (17) Post-ERCP:2 (14)/1 (6)/2 (7) Other:5 (36)/8 (44)/19 (63)	PCD+VARD/ ETD+ETN/ON	13 (93)/ 13 (72)/ 25 (83)	39 (15–184)/ 54 (8–194)/ 11 (0–77)						

### Risk of Bias and Methodological Quality

**Figure 2:**
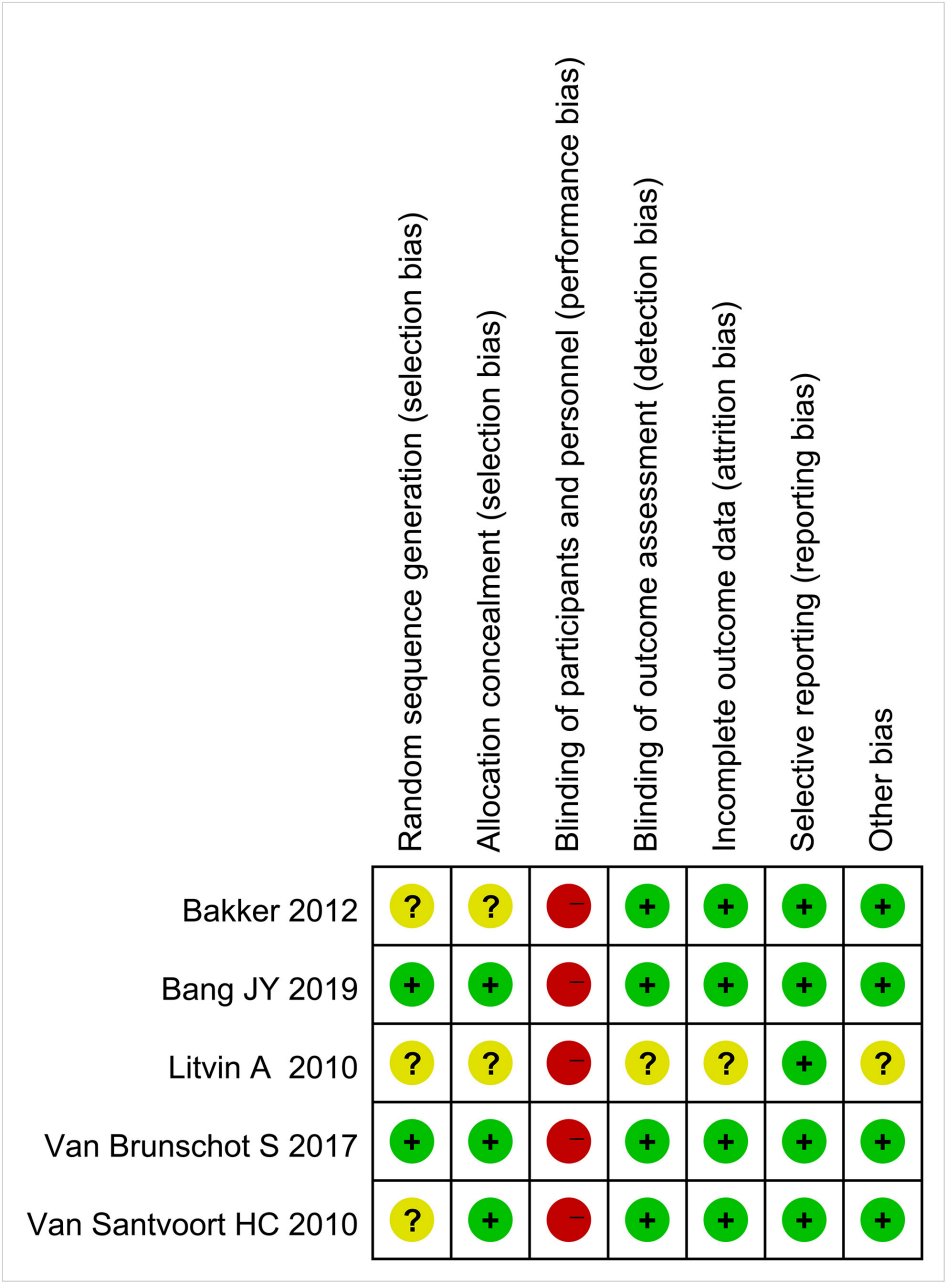
Risk of bias.

Figure 2 shows the risk of bias of included RCTs. The quality evaluation of RCTs is shown in Table 3, and the quality evaluation of cohort studies are shown in Table 4.

**Table 3:** Assessment of risk of bias of included RCTs.

**Study**	**Random sequence generation**	**Adequate allocation concealment**	**Blinding of participants and personnel**	**Blinding of outcome assessment**	**Incomplete outcome data**	**Selective reporting**	**Other bias**
Bang et al. (5)	Low	Low	High	Low	Low	Low	Low
van Brunschot et al. (6)	Low	Low	High	Low	Low	Low	Low
Bakker et al. (7)	Unclear	Unclear	High	Low	Low	Low	Low
Litvin and Khokha (16)	Unclear	Unclear	High	Unclear	Unclear	Low	Unclear
Van Santvoort et al. (8)	Unclear	Low	High	Low	Low	Low	Low

**Table 4:** Assessment of quality of included PCS or RCS.

**Study**	**selection**				**Comparability**		**Outcome**		
	**Representativeness of treated arm**	**Selection of the comparative treatment arm(s)**	**Ascertainment of the treatment regimen**	**Demonstration that outcome of interest was not present at start of study**	**Comparability between patients in different treatment arms: main factor**	**Comparability between patients in different treatment arms: secondary factor**	**Assessment of outcome with independency**	**Adequacy of follow-up length (to assess outcome)**	**Lost to follow-up acceptable (<10% and reported)**
He et al. (10)	Yes	Yes	Yes	Yes	Yes	No	No	Yes	Yes
Kumar et al. (11)	Yes	Yes	Yes	Yes	Yes	No	No	Yes	Yes
Khreiss et al. (12)	Yes	Yes	Yes	Yes	Yes	No	No	Yes	Yes
Woo et al. (13)	Yes	Yes	Yes	Yes	Yes	No	No	No	Yes
Tan et al. (14)	Yes	Yes	Yes	Yes	Yes	No	No	No	Yes
Bausch et al. (15)	Yes	Yes	Yes	Yes	Yes	No	No	Yes	Yes

### Outcomes

#### Major Complications or Death

**Figure 3:**
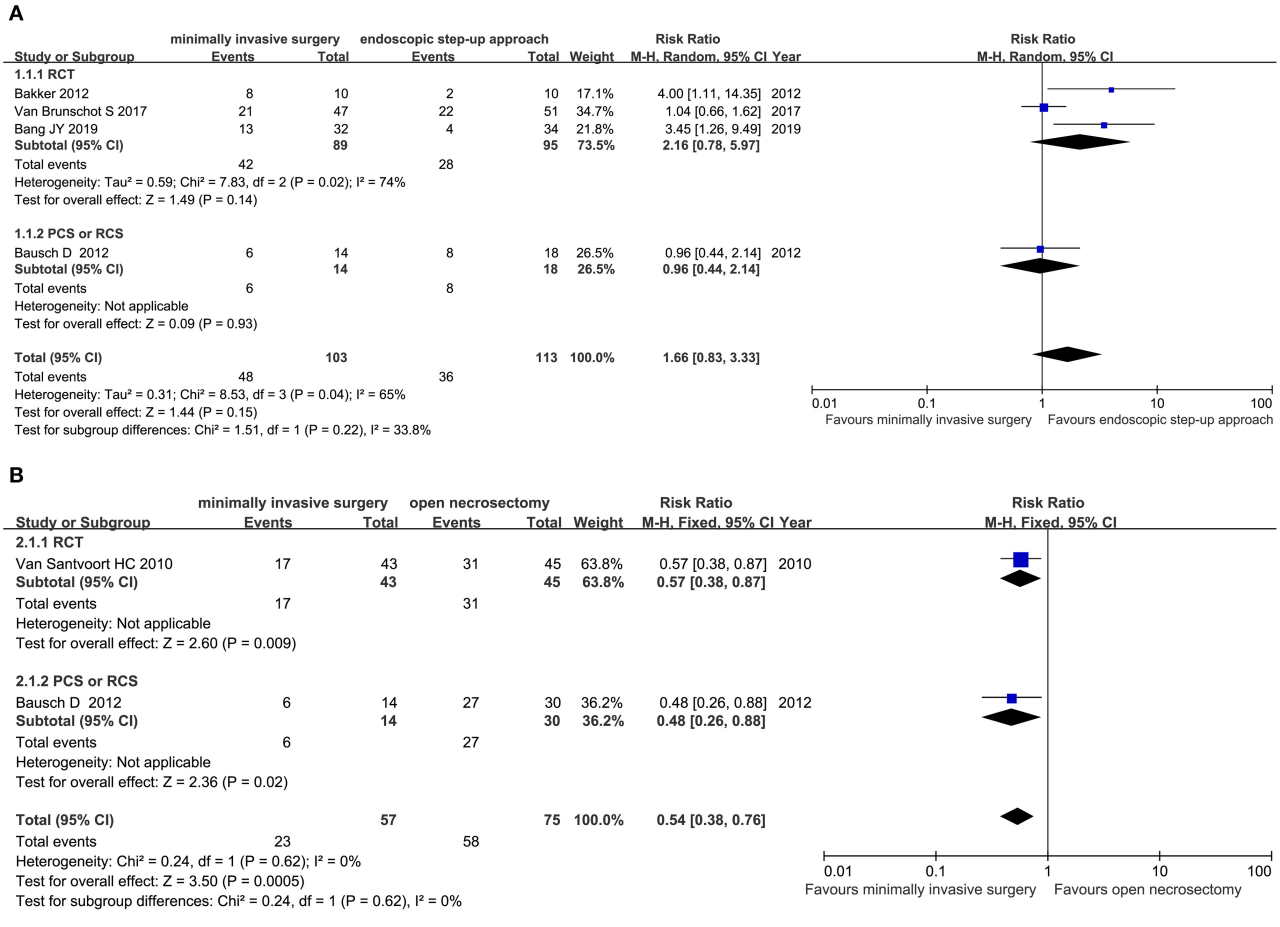
Forest plots for the major complications or death rates. **(A)** Between minimally invasive surgery and endoscopic step-up approach. **(B)** Between minimally invasive surgery and open necrosectomy. M-H, Mantel–Haenszel test; Random, a random effects model; CI, confidence intervals.

Four studies (3 RCT and 1 CS) analyzed the major complications or death rates of minimally invasive surgery and endoscopic step-up approach. Results showed that there was no significant difference in major complications or death between the minimally invasive surgery group and the endoscopic step-up approach group (RR = 1.66, 95% CI: 0.83–3.33, *P* = 0.15; Figure 3A).

Two studies (1 RCT, 1 CS) analyzed the major complications or death of minimally invasive surgery and open necrosectomy. The results showed that the major complications or death rate in the minimally invasive surgery group was significantly lower than that in the open necrosectomy group (RR = 0.54, 95% CI: 0.38–0.76, *P* = 0.0005; Figure 3B).

#### Mortality

**Figure 4:**
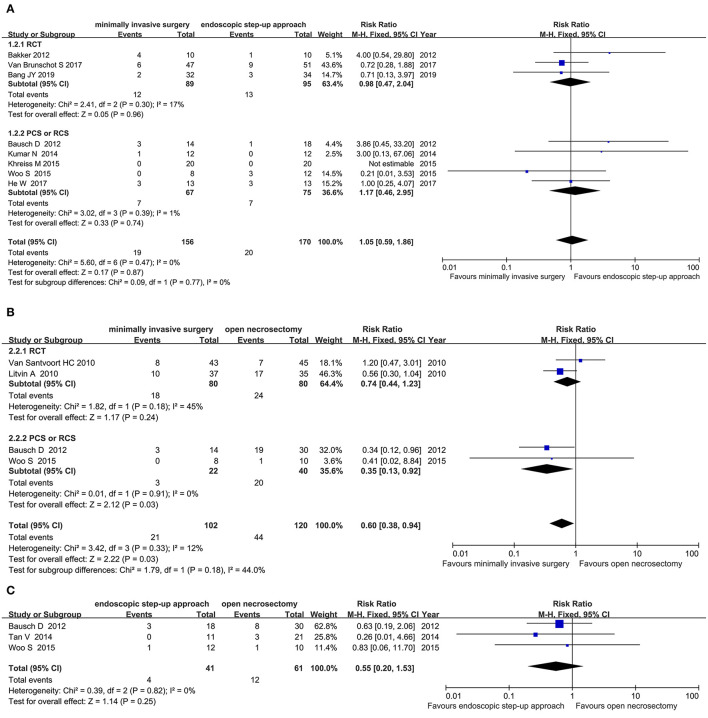
Forest plots for the mortality. **(A)** Between minimally invasive surgery and endoscopic step-up approach. **(B)** Between minimally invasive surgery and open necrosectomy. **(C)** Between endoscopic step-up approach and open necrosectomy. M-H, Mantel–Haenszel test; Fixed, a fixed effects model; CI, confidence intervals.

Eight studies (3 RCT, 5 CS) analyzed the mortality of minimally invasive surgery and endoscopic step-up approach. Results indicated that for mortality, there was no significant difference between the minimally invasive surgery group and endoscopic step-up approach group (RR = 1.05, 95% CI: 0.59–1.86, *P* = 0.87; Figure 4A).

Four studies (2 RCT, 2 CS) analyzed the mortality of minimally invasive surgery and open necrosectomy. The results showed that the mortality rate of the minimally invasive surgery group was significantly lower than that of the open necrosectomy group (RR = 0.60, 95% CI: 0.38–0.94, *P* = 0.03; Figure 4B).

Three cohort studies analyzed the mortality of endoscopic step-up approach and open necrosectomy. The results showed that there was no significant difference in post-operative mortality between the endoscopic step-up approach group and the open necrosectomy group (RR = 0.39, 95% CI: 0.04–3.51, *P* = 0.40; Figure 4C).

#### New-Onset Multiple Organ Failure

**Figure 5:**
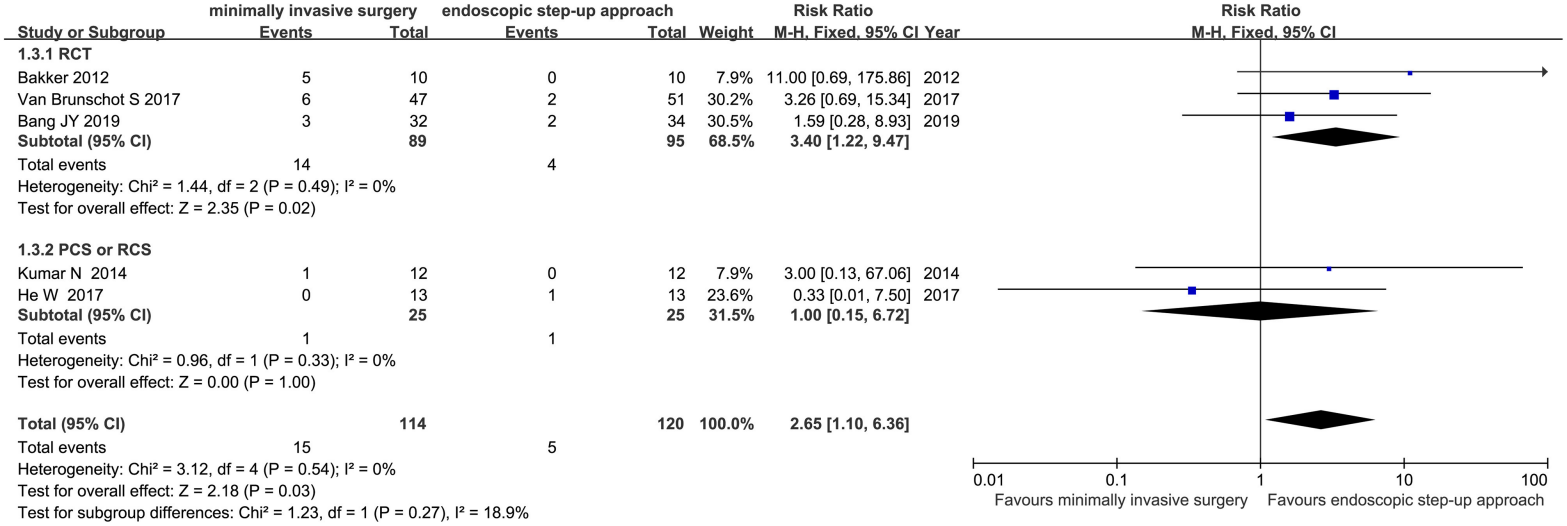
Forest plots for the incidence of new-onset multiple organ failure in patients with minimally invasive surgery and endoscopic step-up approach. M-H, Mantel–Haenszel test; Fixed, a fixed effects model; CI, confidence intervals.

Five studies (3 RCT, 2 CS) analyzed the incidence of new-onset multiple organ failure in patients with minimally invasive surgery and endoscopic step-up approach among the 5 studies. The results showed that the incidence of new-onset multiple organ failure in the endoscopic step-up approach group was significantly lower than that in the minimally invasive surgery group (RR = 2.65, 95% CI: 1.10–6.36, *P* = 0.03; Figure 5).

#### Enterocutaneous Fistula

**Figure 6:**
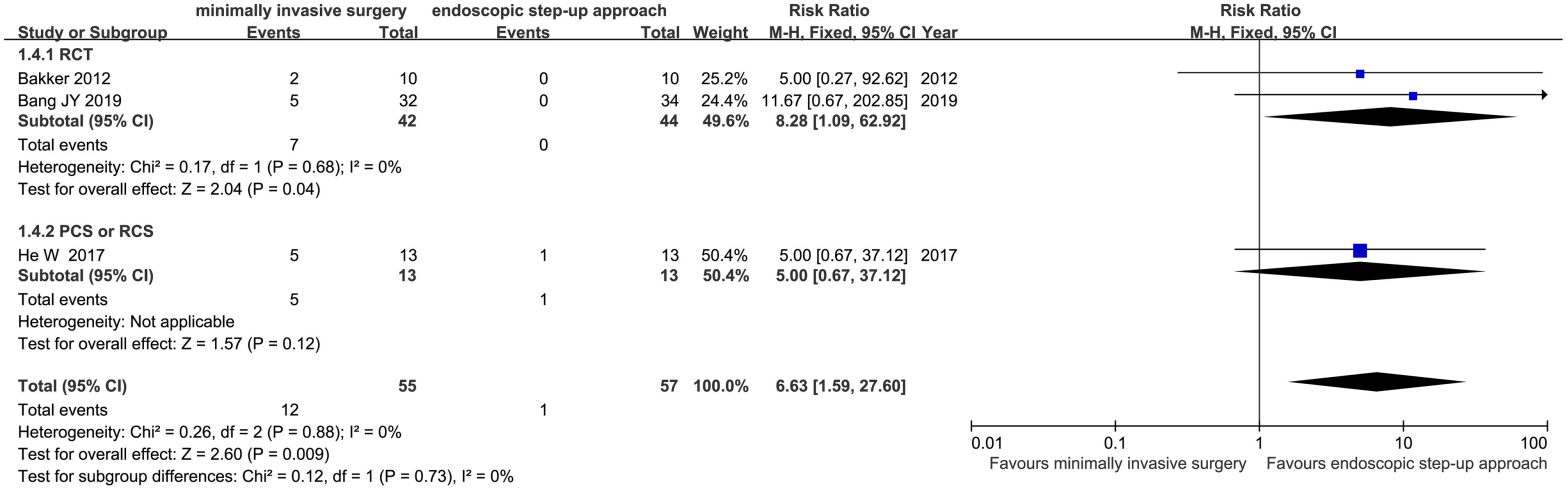
Forest plots for the incidence of intestinal fistula after minimally invasive surgery and endoscopic step-up approach. M-H, Mantel–Haenszel test; Fixed, a fixed effects model; CI, confidence intervals.

Three studies (2 RCT, 1 CS) analyzed the incidence of intestinal fistula after minimally invasive surgery and endoscopic step-up approach. The results showed that the incidence of enterocutaneous fistula in the endoscopic step-up approach group was significantly lower than that in the minimally invasive surgery group (RR = 6.63, 95% CI: 1.59–27.60, *P* = 0.009; Figure 6).

#### Pancreatic-Cutaneous Fistula

**Figure 7:**
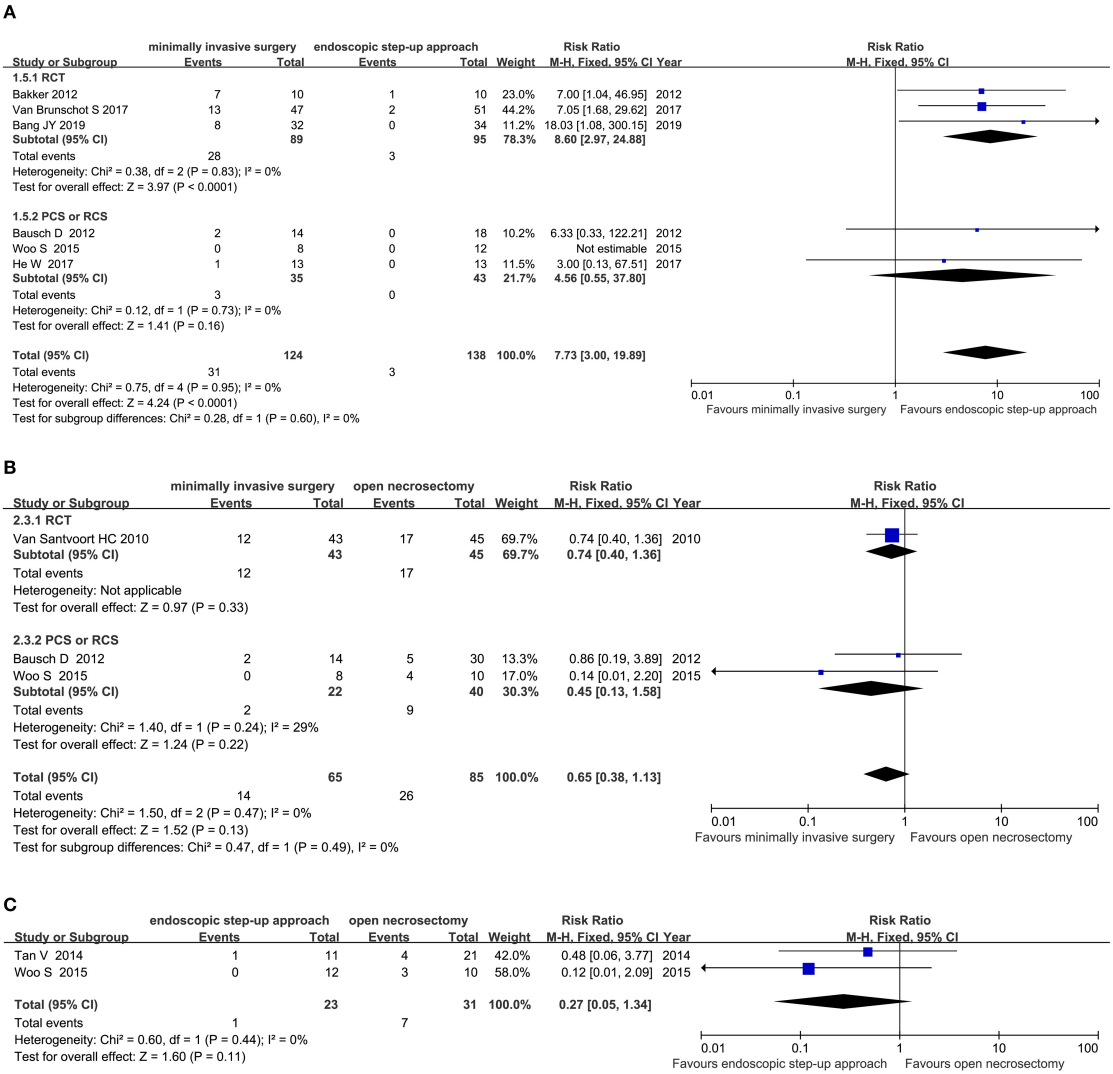
Forest plots for the incidence of pancreatic fistulas. **(A)** Between minimally invasive surgery and endoscopic step-up approach. **(B)** Between minimally invasive surgery and open necrosectomy. **(C)** Between endoscopic step-up approach and open necrosectomy M-H, Mantel–Haenszel test; Fixed, a fixed effects model; CI, confidence intervals.

Six studies (3 RCT, 3 CS) analyzed the incidence of pancreatic fistulas after minimally invasive surgery and endoscopic step-up approach. The results showed that the incidence of pancreatic-cutaneous fistula in the endoscopic step-up approach group was significantly lower than that in the minimally invasive surgery group (RR = 7.73, 95% CI: 3.00–19.89, *P* < 0.0001; Figure 7A).

Three studies (1 RCT, 2 CS) analyzed the incidence of pancreatic fistula after minimally invasive surgery and open necrosectomy. The results showed that there was no significant difference in the incidence of pancreatic-cutaneous fistula between the minimally invasive surgery group and the open necrosectomy group (RR = 0.65, 95% CI: 0.38–1.13, *P* = 0.13; Figure 7B).

Three cohort studies analyzed the incidence of pancreatic fistula after endoscopic step-up approach and open necrosectomy. The results showed that the incidence of pancreatic-cutaneous fistula in the endoscopic step-up approach group was significantly lower than that in the open necrosectomy group (RR = 0.11, 95% CI: 0.02–0.58, *P* = 0.009; Figure 7C).

#### Intra-Abdominal Bleeding

**Figure 8:**
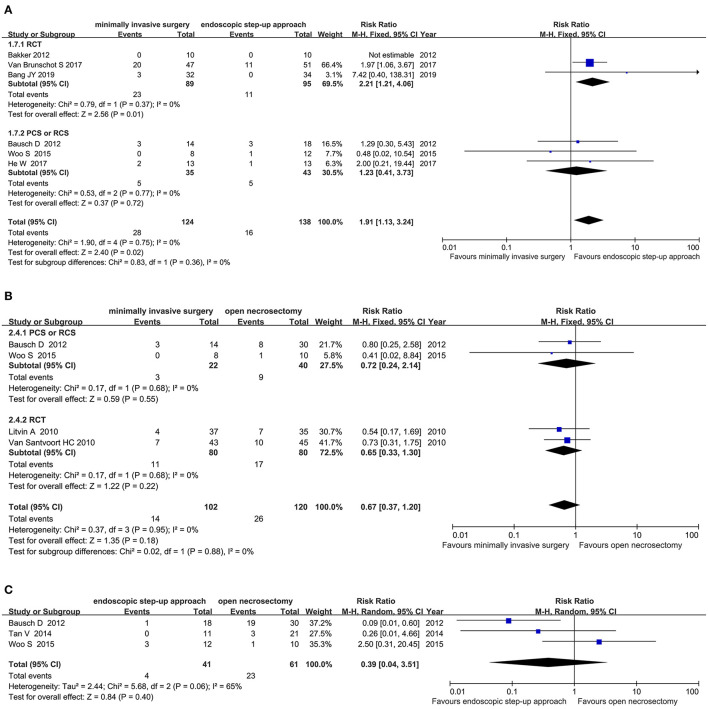
Forest plots for the incidence of intra-abdominal bleeding. **(A)** Between minimally invasive surgery and endoscopic step-up approach. **(B)** Between minimally invasive surgery and open necrosectomy. **(C)** Between endoscopic step-up approach and open necrosectomy. M-H, Mantel–Haenszel test; Fixed, a fixed effects model; CI, confidence intervals.

Six studies (3RCT, 3 CS) analyzed the incidence of intra-abdominal bleeding after minimally invasive surgery and endoscopic step-up approach. The results showed that the incidence of intra-abdominal bleeding in the endoscopic step-up approach group was significantly lower than that in the minimally invasive surgery group (RR = 1.91, 95% CI: 1.13–3.24, *P* = 0.02; Figure 8A).

Four studies (2 RCT, 2 CS) analyzed the incidence of intra-abdominal bleeding after minimally invasive surgery and open necrosectomy. The results showed that there was no significant difference in the incidence of post-operative intra-abdominal bleeding between the minimally invasive surgery group and the open necrosectomy group (RR = 0.67, 95% CI: 0.37–1.20, *P* = 0.18; Figure 8B).

Three cohort studies analyzed the incidence of intra-abdominal bleeding after endoscopic step-up approach and open necrosectomy. The results showed that there was no significant difference in the incidence of intra-abdominal bleeding between the endoscopic step-up approach group and the open necrosectomy group (RR = 0.55, 95% CI: 0.20–1.53, *P* = 0.25; Figure 8C).

#### Length of Hospital Stay

**Figure 9:**
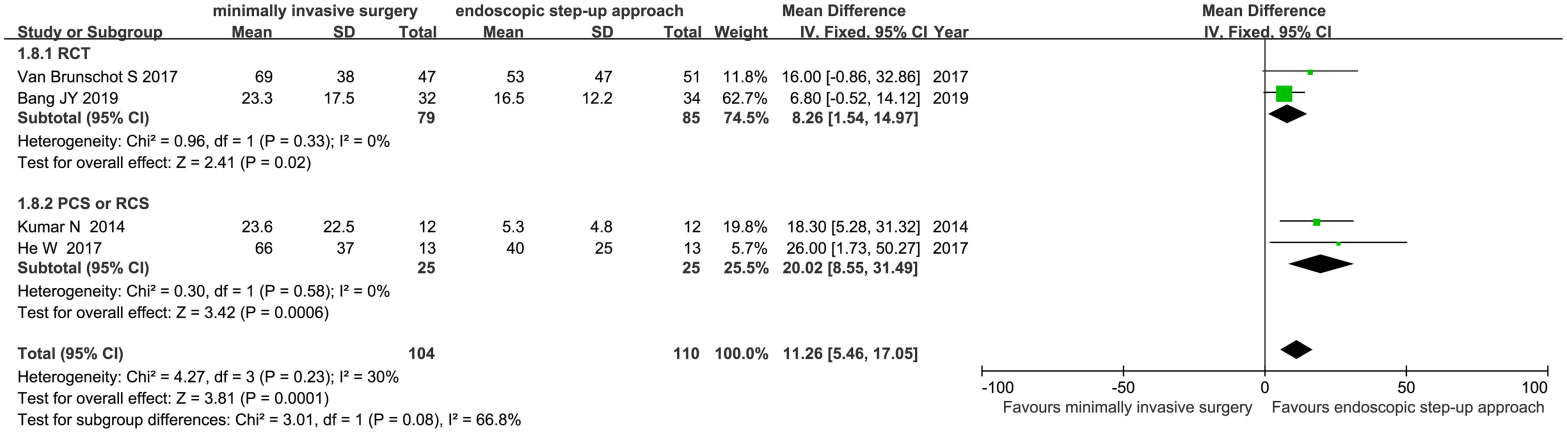
Forest plots for the length of hospital for minimally invasive surgery and endoscopic step-up approach. IV, inverse variance; Fixed, a fixed effects model; CI, confidence intervals.

Four studies (2 RCT, 2 CS) analyzed the length of hospital for minimally invasive surgery and endoscopic step-up approach. The results showed that the hospital stay of endoscopic step-up approach group was significantly shorter than that of the minimally invasive surgery group (MD = 11.26, 95% CI: 5.46–17.05, *P* = 0.0001; Figure 9).

#### Length of ICU Stay

**Figure 10:**
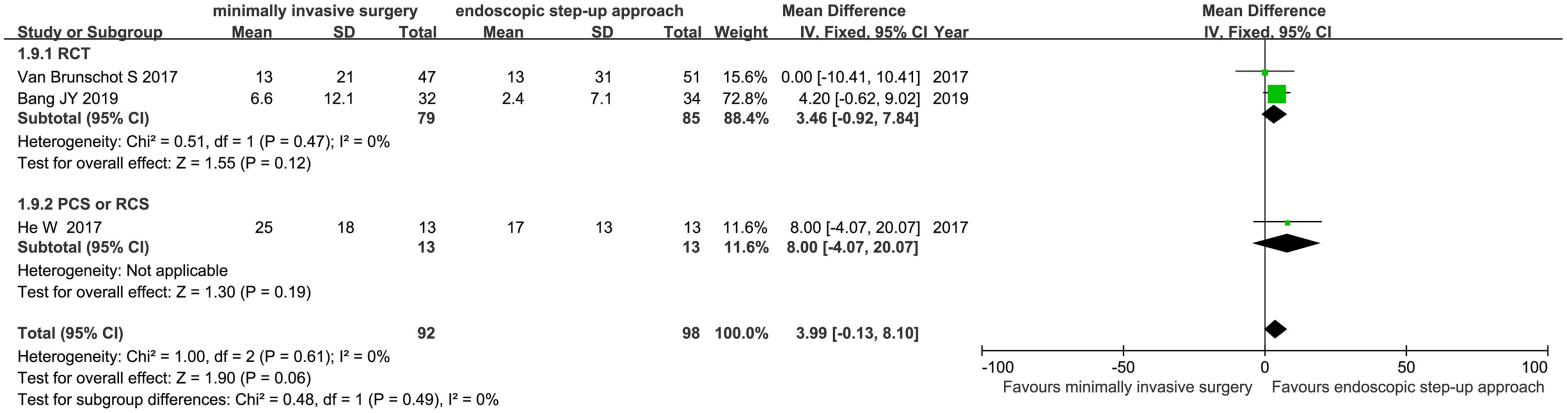
Forest plots for ICU duration of minimally invasive surgery and endoscopic step-up approach. IV, inverse variance; Fixed, a fixed effects model; CI, confidence intervals.

Three studies (2 RCT and 1 CS) analyzed the ICU duration of minimally invasive surgery and endoscopic step-up approach. The results showed that there was no significant difference in length of ICU stay between the minimally invasive surgery treatment group and the endoscopic step-up approach treatment group (MD = 3.99, 95% CI: −0.13 to 8.0, *P* = 0.06; Figure 10).

#### Endocrine Pancreatic Insufficiency

**Figure 11:**
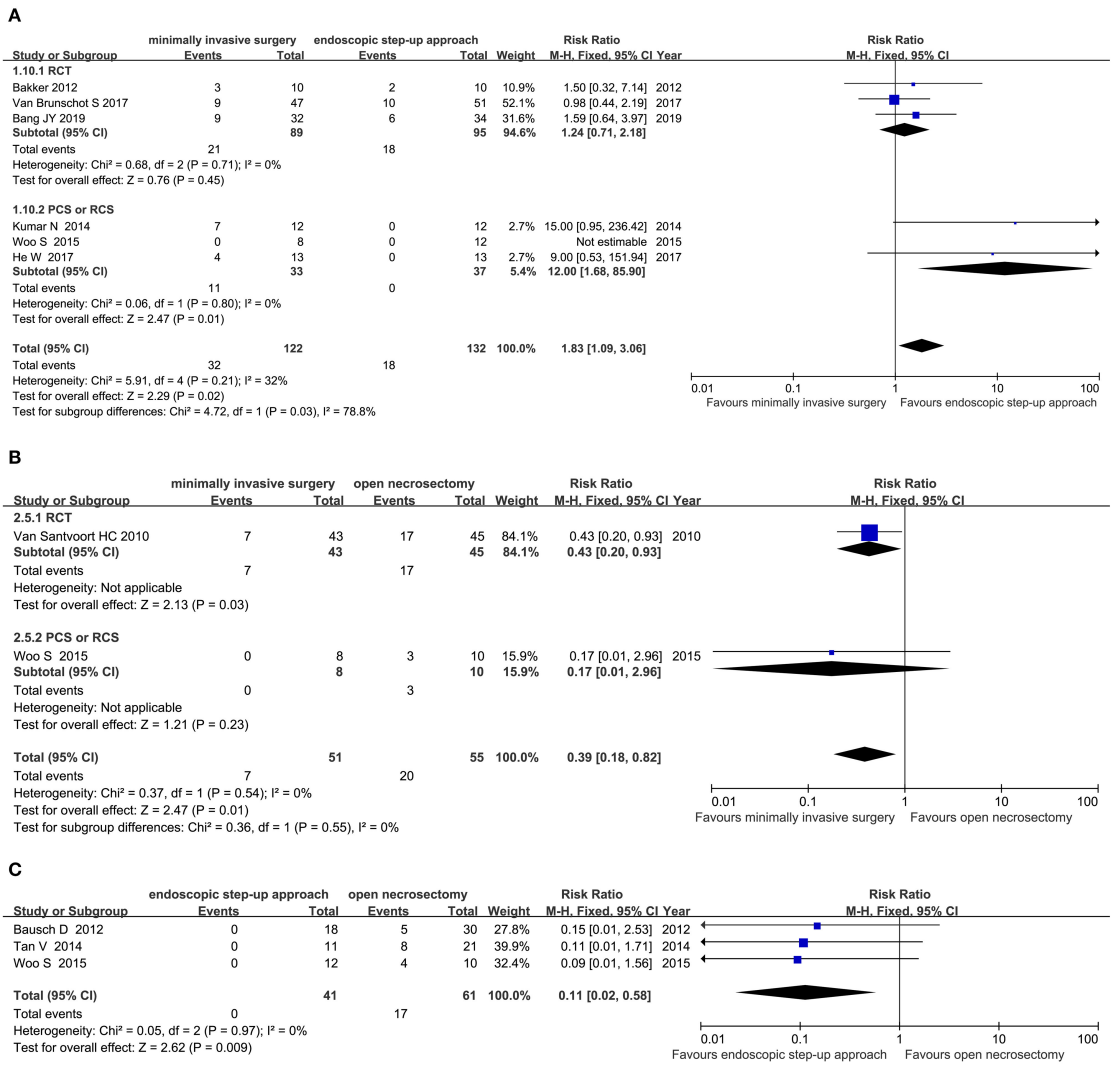
Forest plots for the incidence of endocrine pancreatic insufficiency. **(A)** Between minimally invasive surgery and endoscopic step-up approach. **(B)** Between minimally invasive surgery and open necrosectomy. **(C)** Between endoscopic step-up approach and open necrosectomy. M-H, Mantel–Haenszel test; Fixed, a fixed effects model; CI, confidence intervals.

Six studies (3 RCT, 3 CS) analyzed the incidence of endocrine pancreatic insufficiency after minimally invasive surgery and endoscopic step-up approach. The results showed that the incidence of endocrine pancreatic insufficiency in the endoscopic step-up approach group was significantly lower than that in the minimally invasive surgery group (RR = 1.83, 95% CI: 1.9–3.16, *P* = 0.02; Figure 11A).

Two studies (1 RCT and 1 CS) analyzed the incidence of endocrine pancreatic insufficiency after minimally invasive surgery and open necrosectomy. The results showed that the incidence of endocrine pancreatic insufficiency in the minimally invasive surgery group was significantly lower than that in the open necrosectomy group (RR = 0.39, 95% CI: 0.18–0.82, *P* = 0.01; Figure 11B).

Two cohort studies analyzed the incidence of endocrine pancreatic insufficiency after endoscopic step-up approach and open necrosectomy. The results showed that there was no significant difference in post-operative endocrine pancreatic insufficiency rate between the endoscopic step-up approach treatment group and the open necrosectomy treatment group (RR = 0.27, 95% CI: 0.05–1.34, *P* = 0.11; Figure 11C).

#### Exocrine Pancreatic Insufficiency

**Figure 12:**
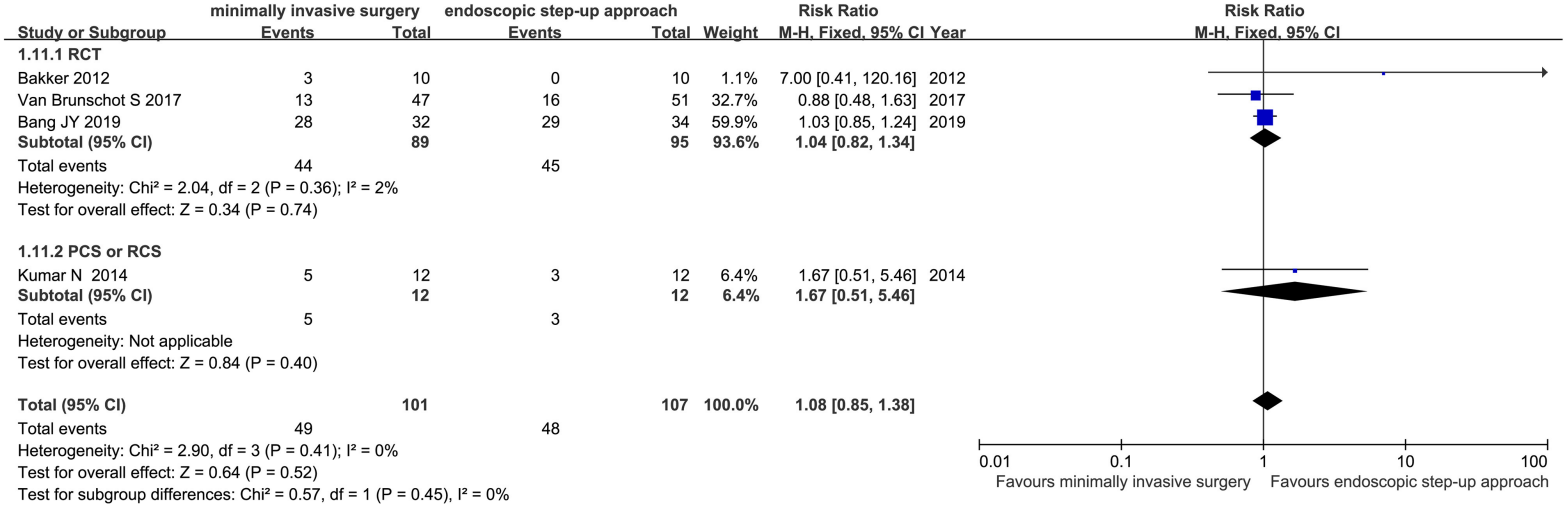
Forest plots for the incidence of exocrine pancreatic insufficiency after minimally invasive surgery and endoscopic step-up approach. M-H, Mantel–Haenszel test; Fixed, a fixed effects model; CI, confidence intervals.

Four studies (3 RCT, 1 CS) analyzed the incidence of exocrine pancreatic insufficiency after minimally invasive surgery and endoscopic step-up approach. The results showed that there was no significant difference in the incidence of post-operative exocrine pancreatic insufficiency between the minimally invasive surgery treatment group and the endoscopic step-up approach treatment group (RR = 1.08, 95% CI: 0.85–1.38, *P* = 0.52; Figure 12).

#### Publication Bias

The funnel plot results showed no publication bias (additional file).

## Discussion

Outcomes of this meta-analysis showed that the major complications or death and mortality of minimally invasive surgery and endoscopic step-up approach are similar, and there is no significant statistical difference. However, endoscopic step-up approach can significantly reduce the incidence of new-onset multiple organ failure, enterocutaneous fistula, pancreatic-cutaneous fistula, intra-abdominal bleeding, endocrine pancreatic insufficiency, and can shorten the patient's hospital stay. Compared with open necrosectomy, minimally invasive surgery can significantly reduce major complications or death, mortality, and incidence of endocrine pancreatic insufficiency. The incidence of intra-abdominal bleeding and pancreatic-cutaneous fistula was similar. Compared with open necrosectomy surgery, endoscopic step-up approach could reduce the incidence of pancreatic-cutaneous fistula, but there is no significant difference regarding mortality, post-operative intra-abdominal bleeding, and endocrine pancreatic insufficiency.

Endoscopic step-up approach included endoscopic transgastric drainage (ETD) first, with the guide of ultrasound and through posterior wall of the stomach or the duodenum, followed by endoscopic transgastric necrosectomy (ETN). The advantage of endoscopic step-up approach is that it does not require general anesthesia and can be performed while the patient is sedated, and it could be repeated if necessary (17), However, the disadvantage is the limited scope of application and the abscess must be close to stomach. Otherwise, most patients may need repeated ETN to remove all necrotic tissue.

Minimally invasive surgery usually involves percutaneous catheter drainage (PCD) followed by video-assisted retroperitoneal debridement (VARD). PCD should be guided by CT or color ultrasound, and the puncture site is usually the abdominal cavity or the retroperitoneum. Compared with endoscopic step-up approach, the advantage of minimally invasive surgery is that most patients do not require VARD repeatedly, but the disadvantage is that the scope of application is relatively limited, and cavity of necrotic tissue should be close to the body surface (18).

Pancreatic fistula and organ failure were common complications of acute necrotizing pancreatitis. This study found that compared with minimally invasive surgery, endoscopic step-up approach has a lower incidence of pancreatic fistula and new-onset multiple organ failure. The low incidence of pancreatic fistula under endoscopic step-up approach may be related to repeated ENT under ETD. Although the patient has experienced multiple ENTs, it helps the removal of necrotic tissue. Minimally invasive surgery could obtain complete removal when the location of the necrotic tissue is closer to the body surface. The low incidence of new-onset multiple organ failure under endoscopic step-up approach may also be related to the complete removal of necrotic tissue after multiple drainages. Both above methods are superior to open necrosectomy, which was the reason of that it was a gradually replaced therapy in recent years (19).

Death is the most serious complication of acute necrotizing pancreatitis, although evidence showed that the major complication or death in the minimally invasive surgery group is significantly lower than that in the open necrosectomy group, and the difference is statistically significant. However, this result come from a combination of major complications and mortality, and only two articles were included. Therefore, the current evidence indicated that there was no significant difference in post-operative mortality between minimally invasive surgery, endoscopic step-up approach, and open necrosectomy. This means that the above three treatment methods cannot significantly reduce the mortality of acute necrotizing pancreatitis. This may also be caused by insufficient data currently available. Larger samples and multi-center trials are needed for further analysis.

In summary, compared with minimally invasive surgery and open necrosectomy, endoscopic step-up approach could reduce the incidence of some serious complications, such as new-onset multiple organ failure, pancreatic-cutaneous fistula, enterocutaneous fistula, pancreatic-cutaneous fistula, intra-abdominal bleeding, endocrine pancreatic insufficiency, and could significantly shorten the patient's hospital stay, although it cannot reduce the major complications or death and mortality of patients. However, due to the small sample size of the studies included, large sample size and high-quality RCT are needed to verify the efficacy and safety of endoscopic step-up approach.

## Data Availability Statement

The original contributions presented in the study are included in the article/Supplementary Material, further inquiries can be directed to the corresponding author/s.

## Author Contributions

WL had primary responsibility for the final content, and designed the research. JX and XQ conducted the research. XQ and FL analyzed the data. JX and FL wrote the manuscript. All authors read and approved the final manuscript.

## Conflict of Interest

The authors declare that the research was conducted in the absence of any commercial or financial relationships that could be construed as a potential conflict of interest.

## Publisher's Note

All claims expressed in this article are solely those of the authors and do not necessarily represent those of their affiliated organizations, or those of the publisher, the editors and the reviewers. Any product that may be evaluated in this article, or claim that may be made by its manufacturer, is not guaranteed or endorsed by the publisher.
