# Supplementary material for: Comparison of Different Surgical Methods for Necrotizing Pancreatitis: A Meta-Analysis
Source: Front Surg. 2021 Sep 22;8:723605. doi: 10.3389/fsurg.2021.723605 (PMC8493073; doi:10.3389/fsurg.2021.723605)
Supplement: Supplementary file 1 [file Data_Sheet_1.docx]

**Supplementary Materials**

**Comparison of different surgical methods for necrotizing pancreatitis: a meta-analysis**

**minimally invasive surgery vs endoscopic step-up approach**


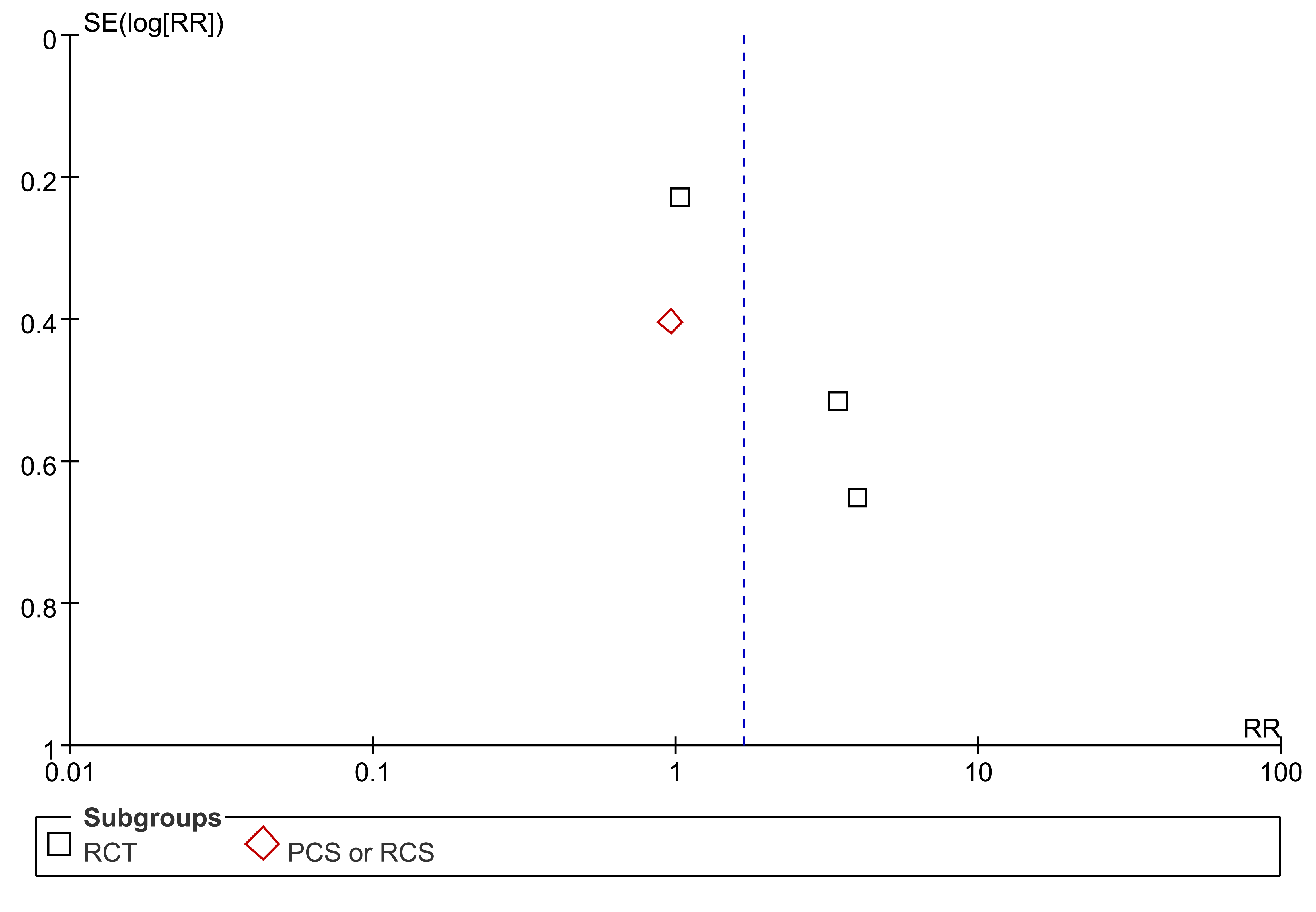


Figure S1 funnel plot of major complications or death


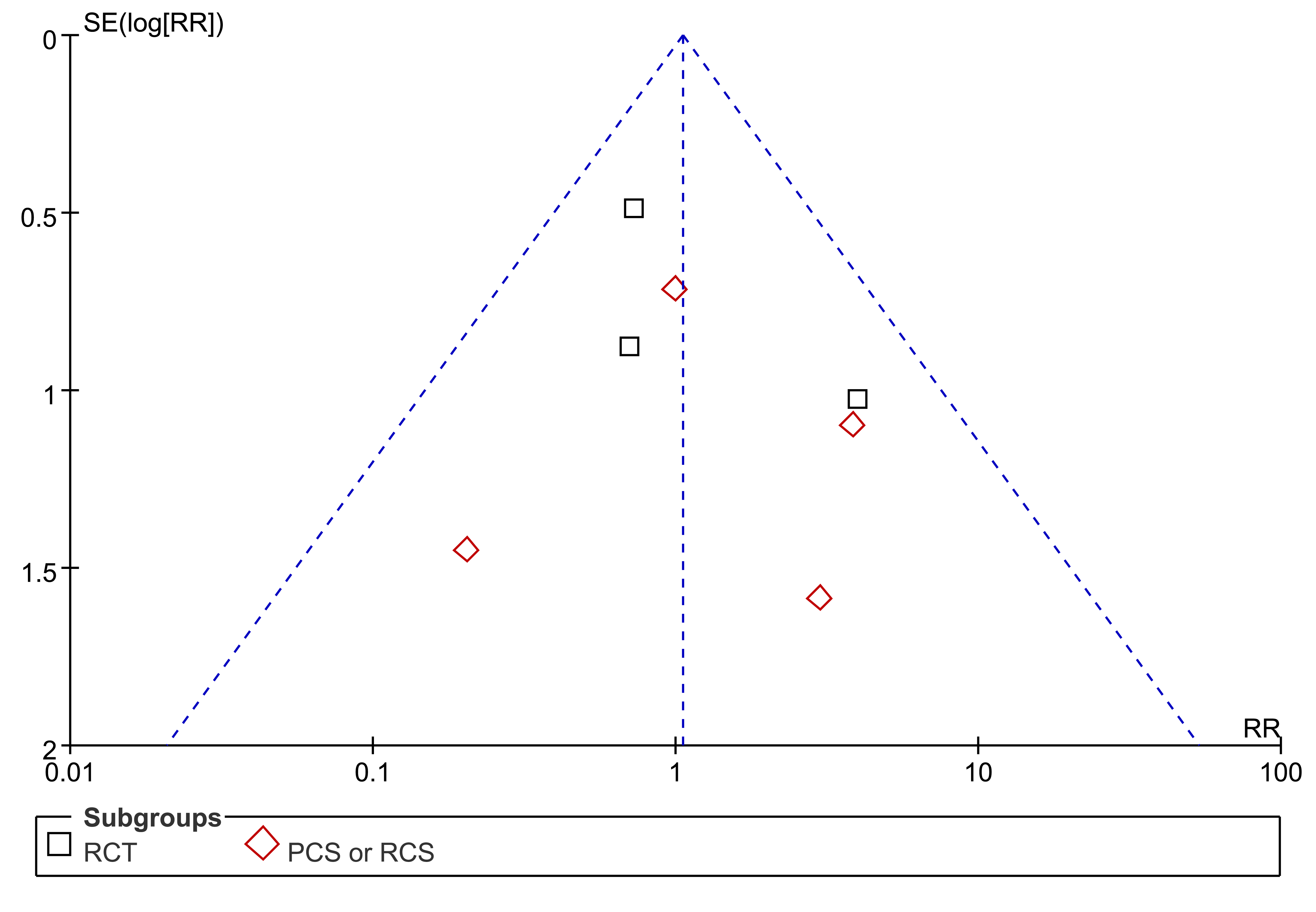


Figure S2 funnel plot of death


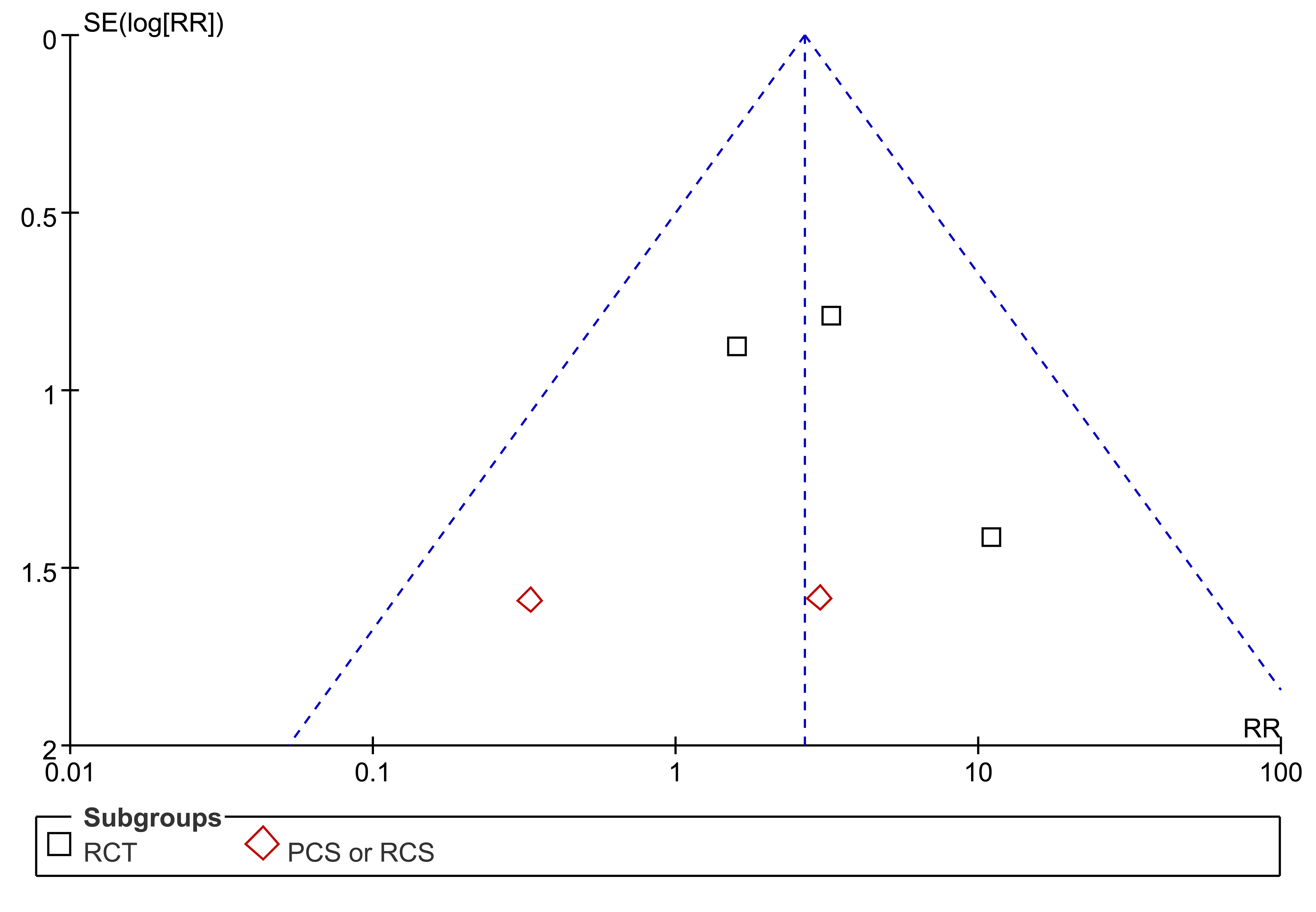


Figure S3 funnel plot of new-onset multiple organ failure


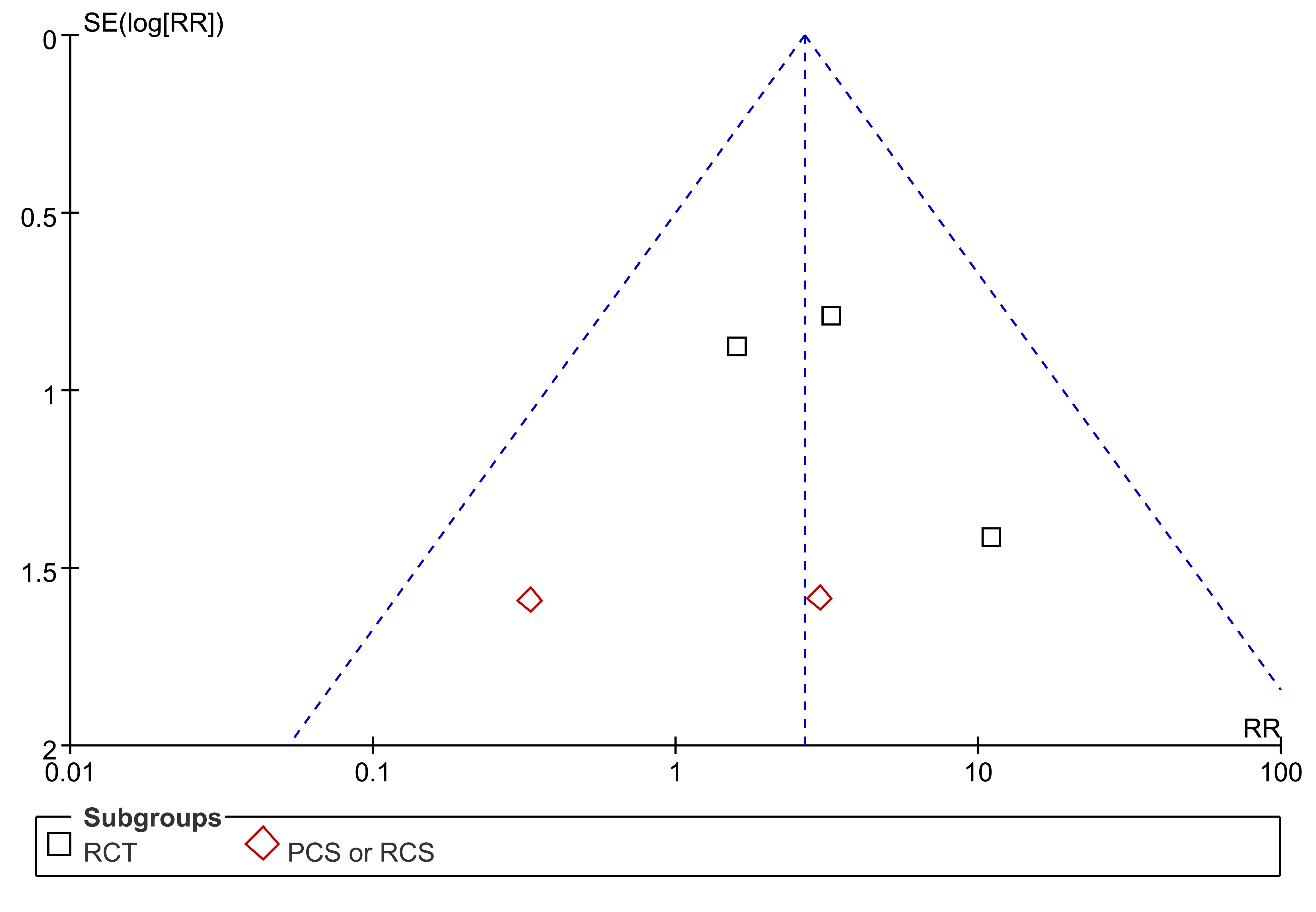


Figure S4 funnel plot of enterocutaneous fistula


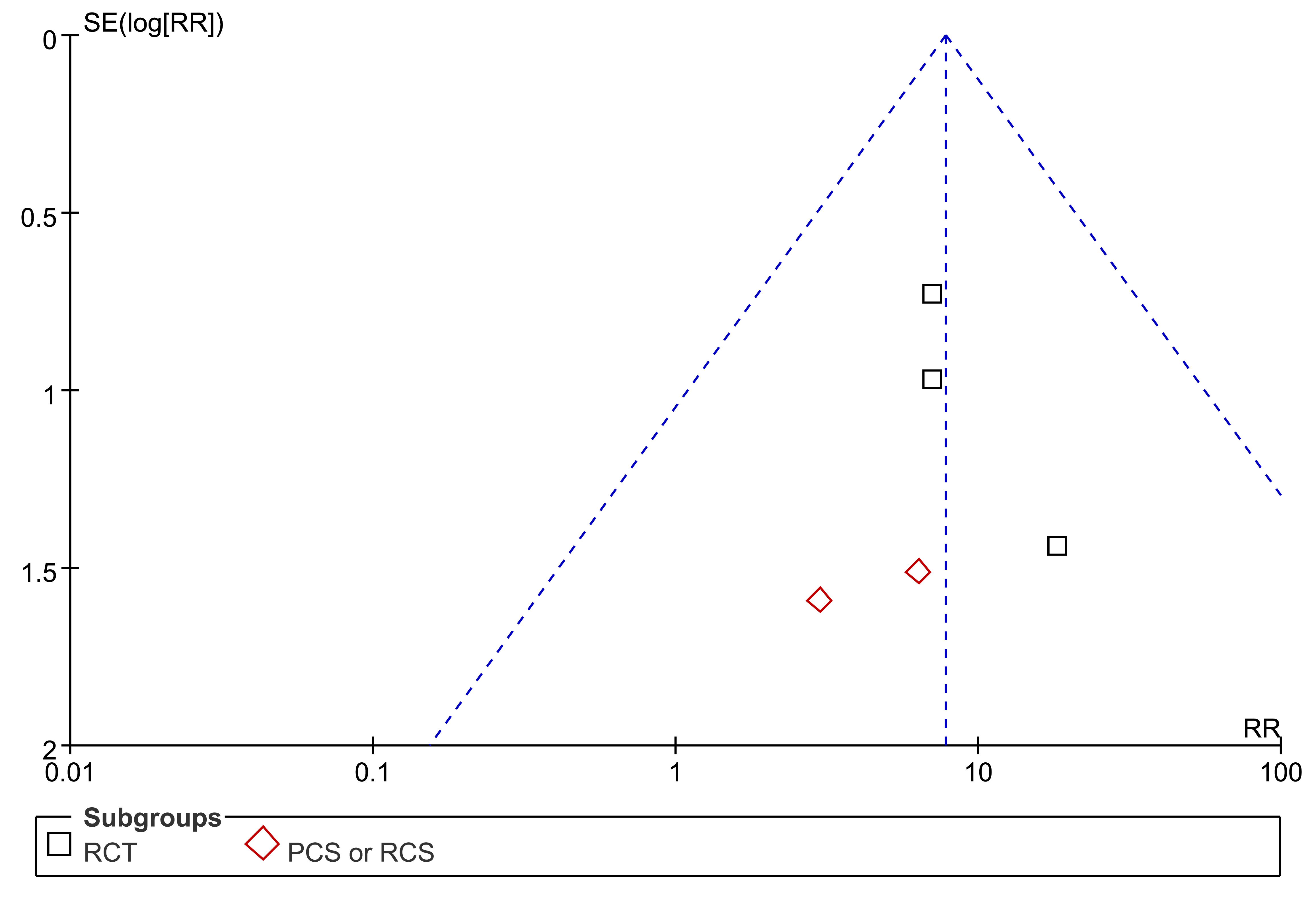


Figure S5 funnel plot of pancreatic-cutaneous fistula


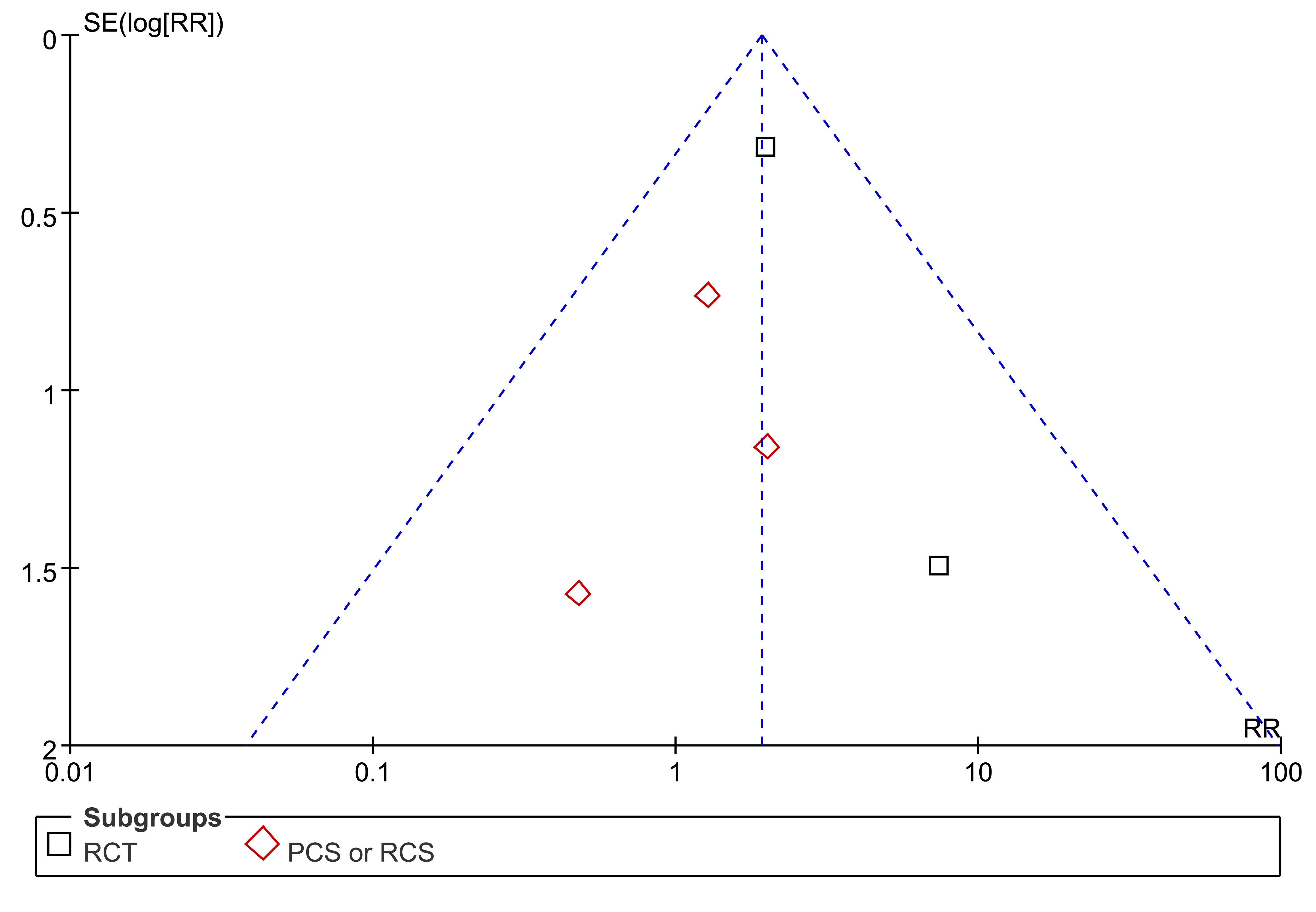


Figure S6 funnel plot of intra-abdominal bleeding


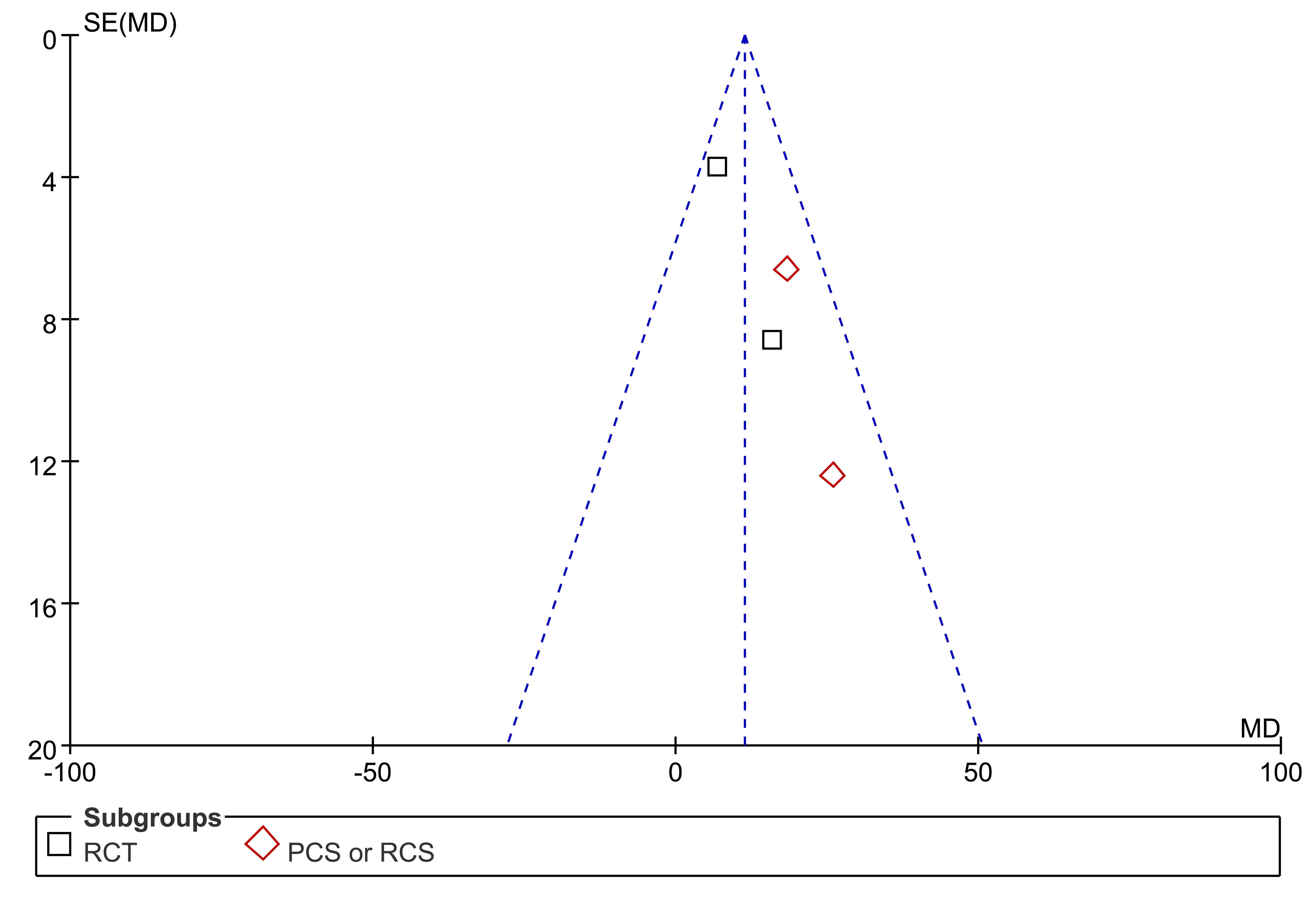


Figure S7 funnel plot of length of hospital stay


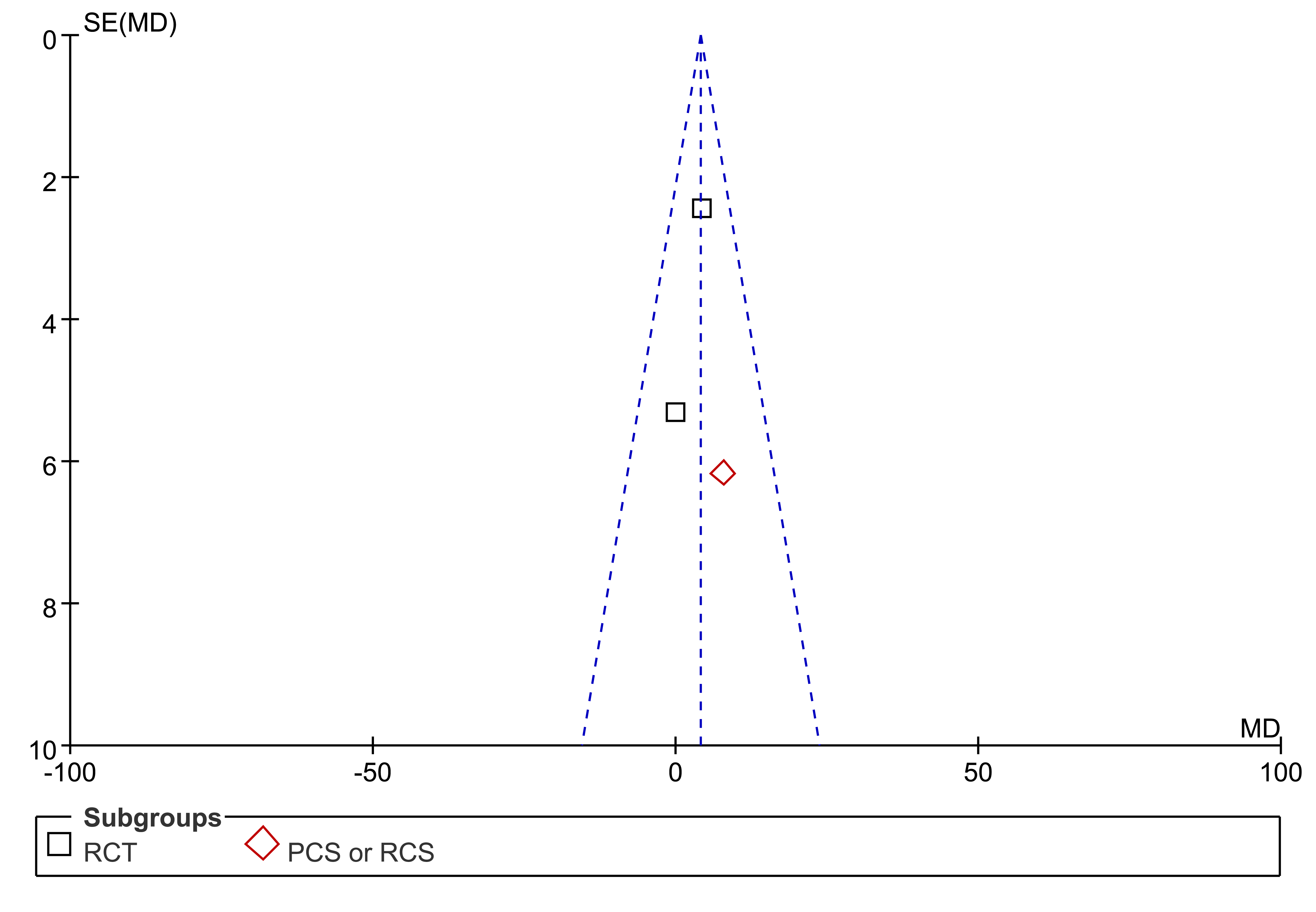


Figure S8 funnel plot of length of ICU stay


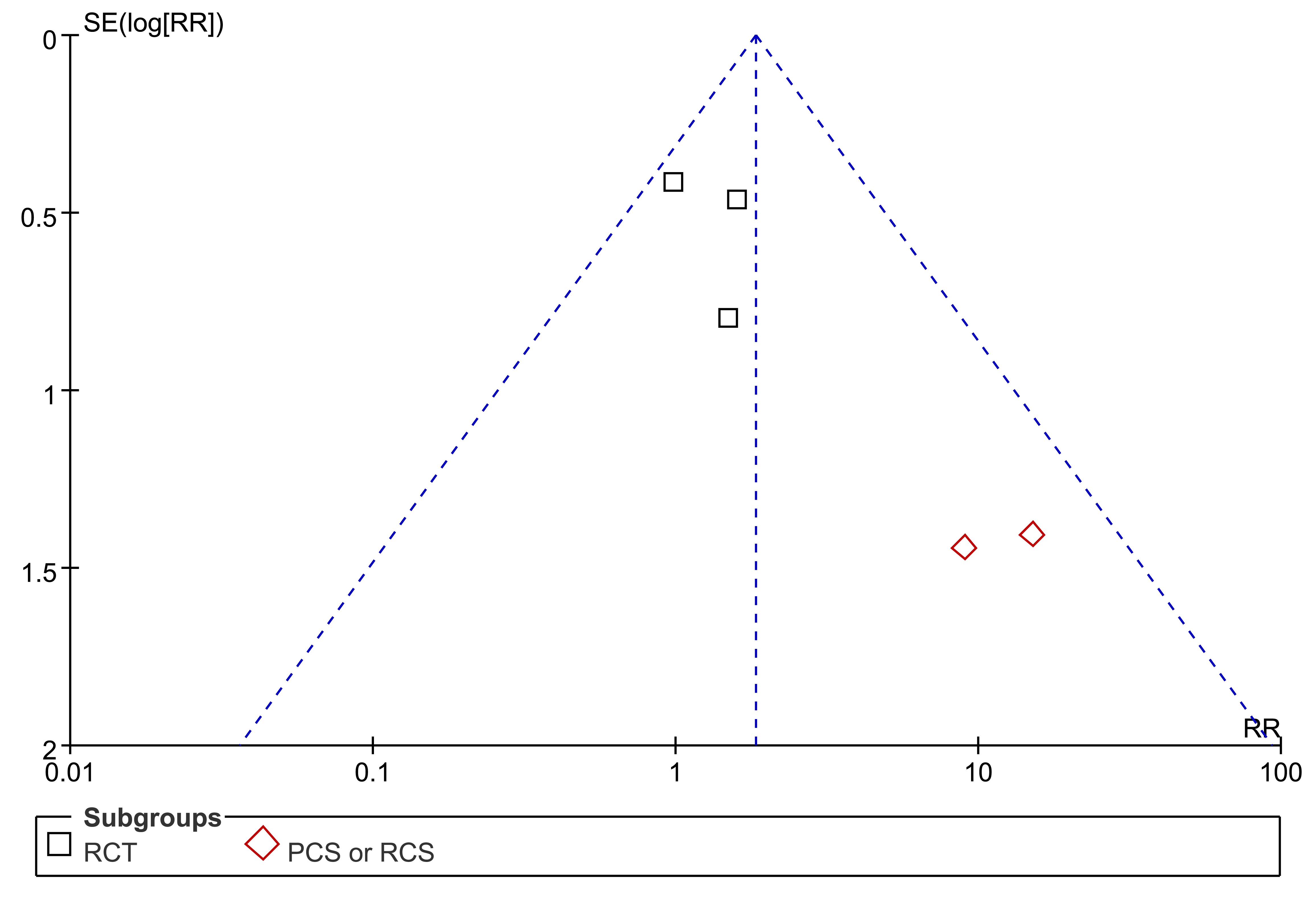


Figure S9 funnel plot of endocrine pancreatic insufficiency


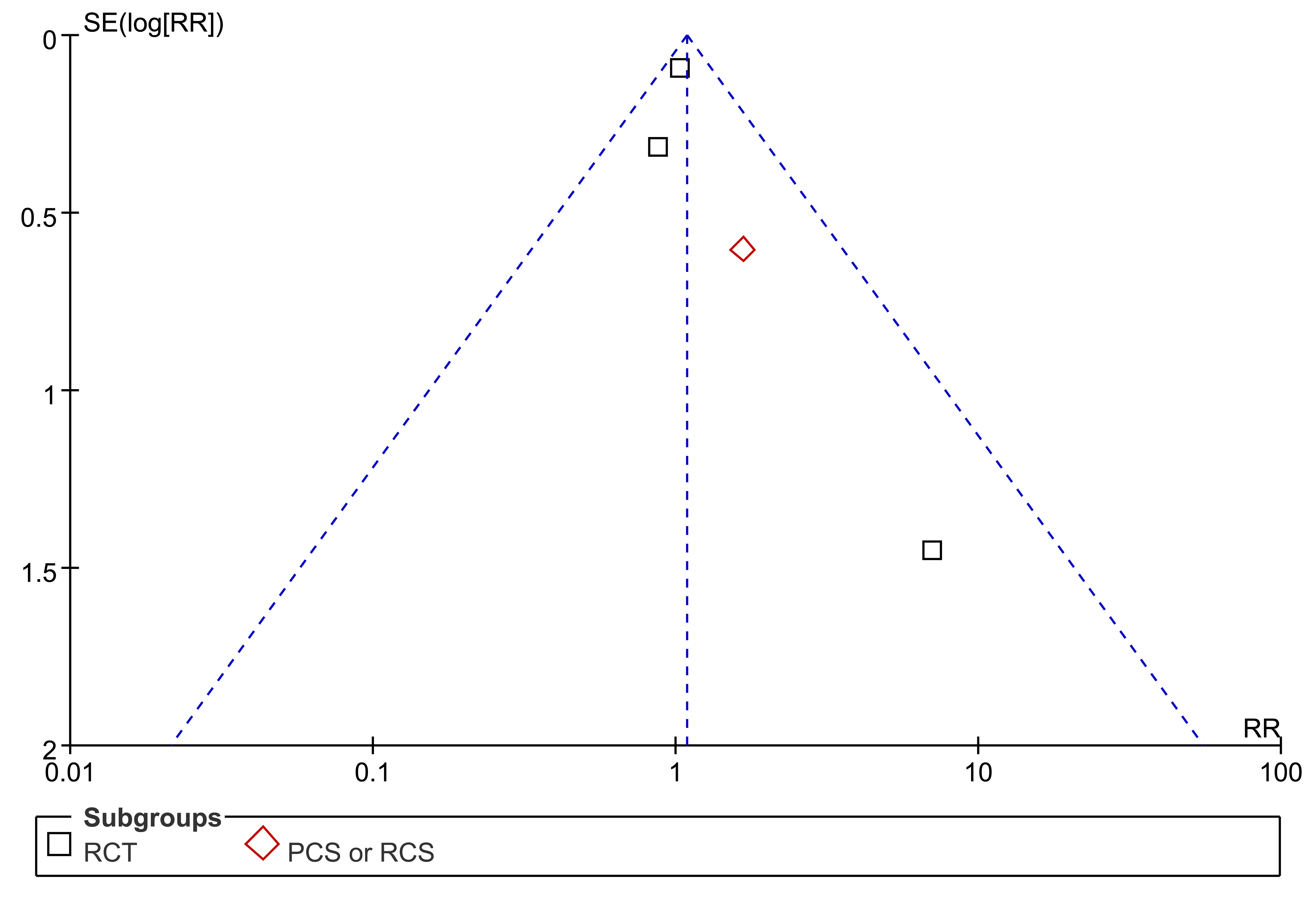


Figure S10 funnel plot of exocrine pancreatic insufficiency

**minimally invasive surgery vs open necrosectomy**


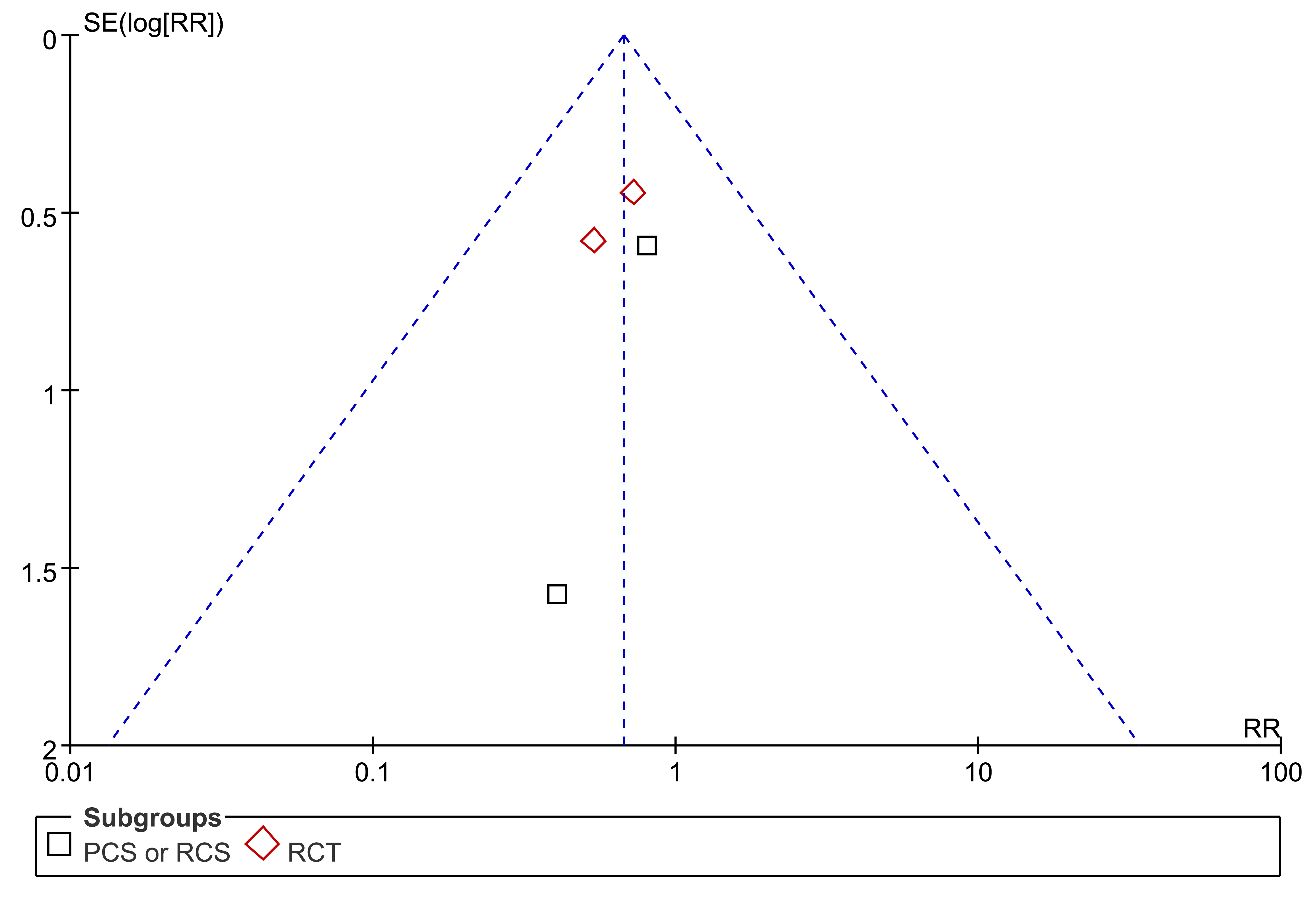


Figure S11 funnel plot of major complications or death


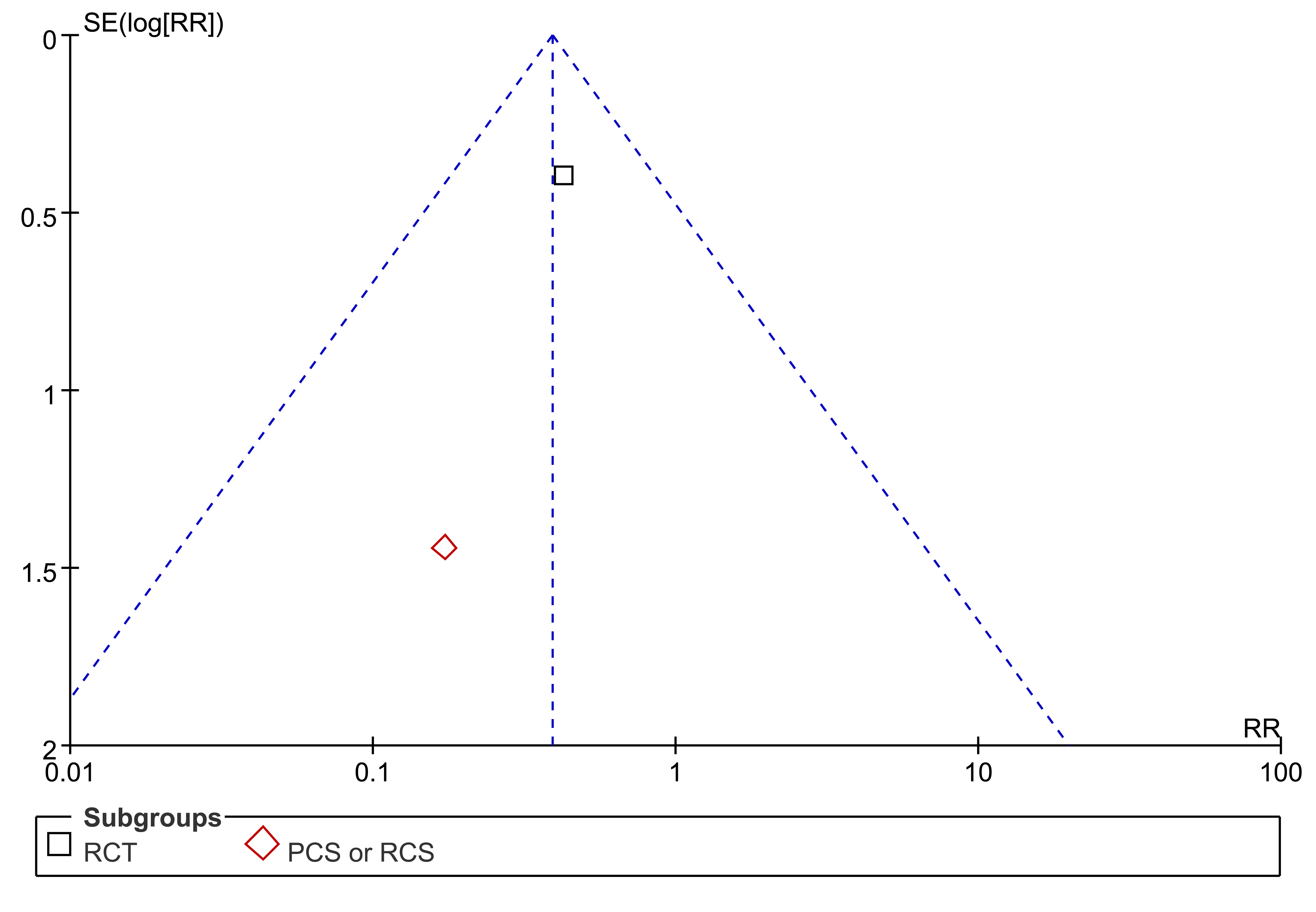


Figure S12 funnel plot of death


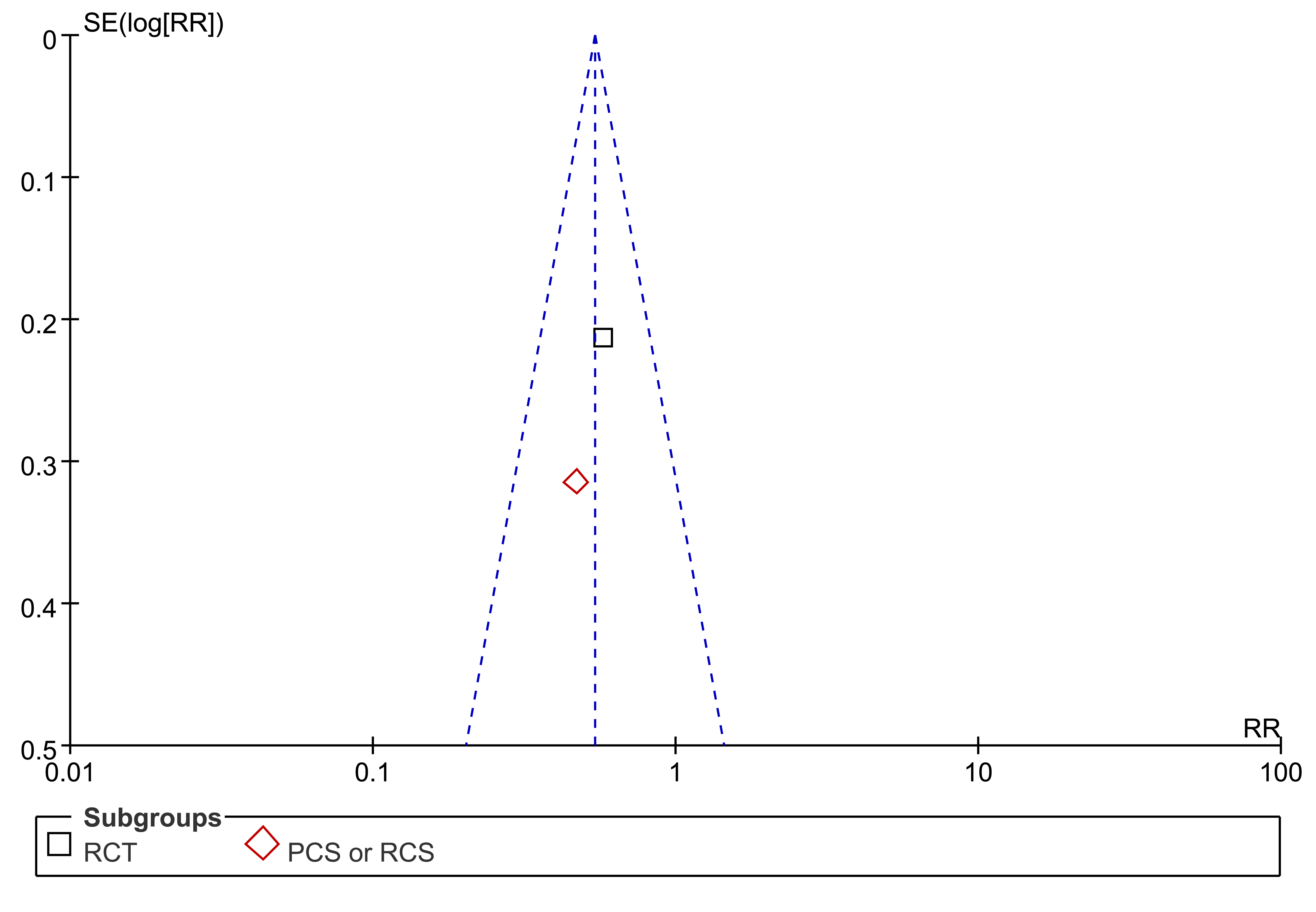


Figure S13 funnel plot of enterocutaneous fistula


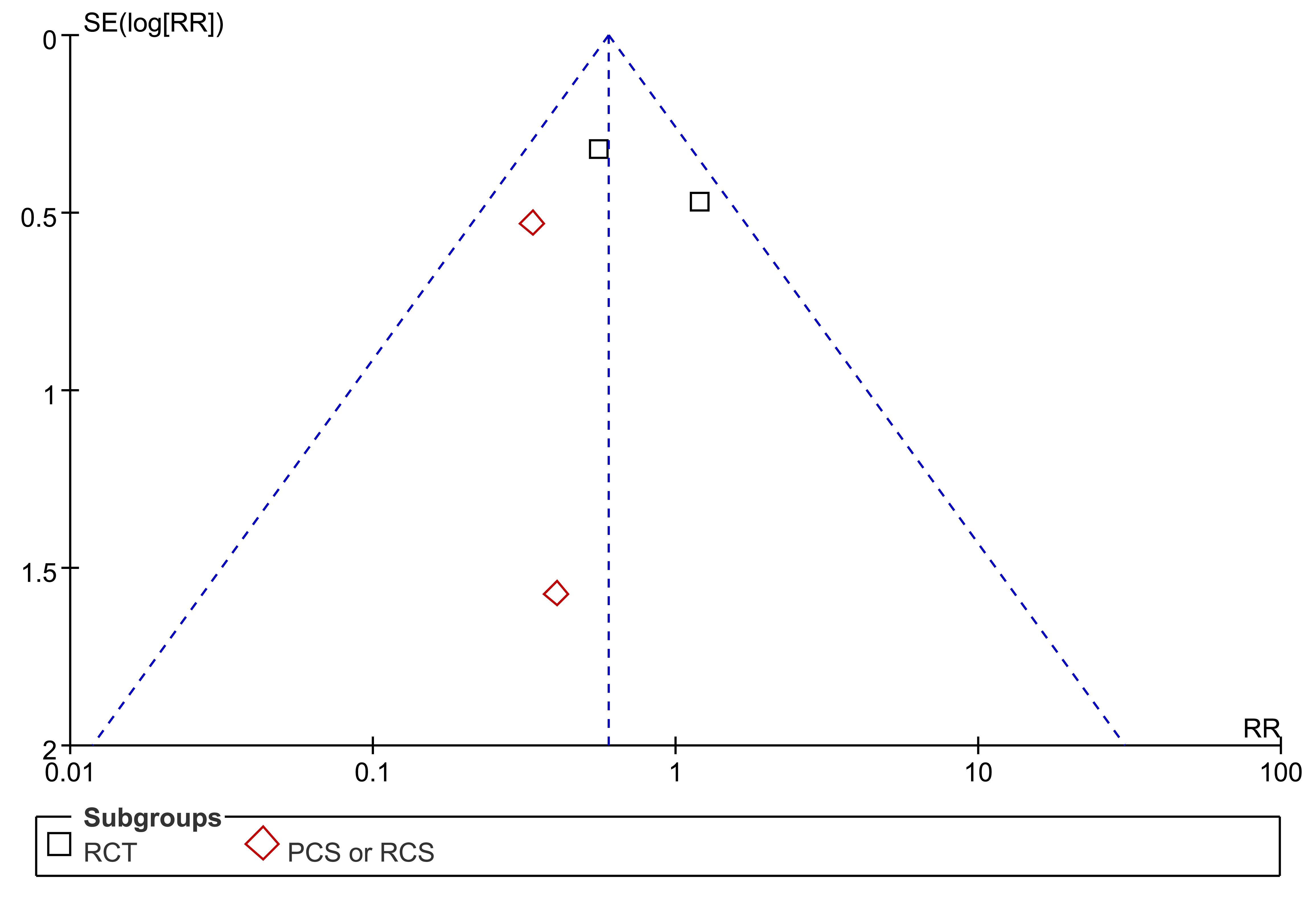


Figure S14 funnel plot of pancreatic-cutaneous fistula


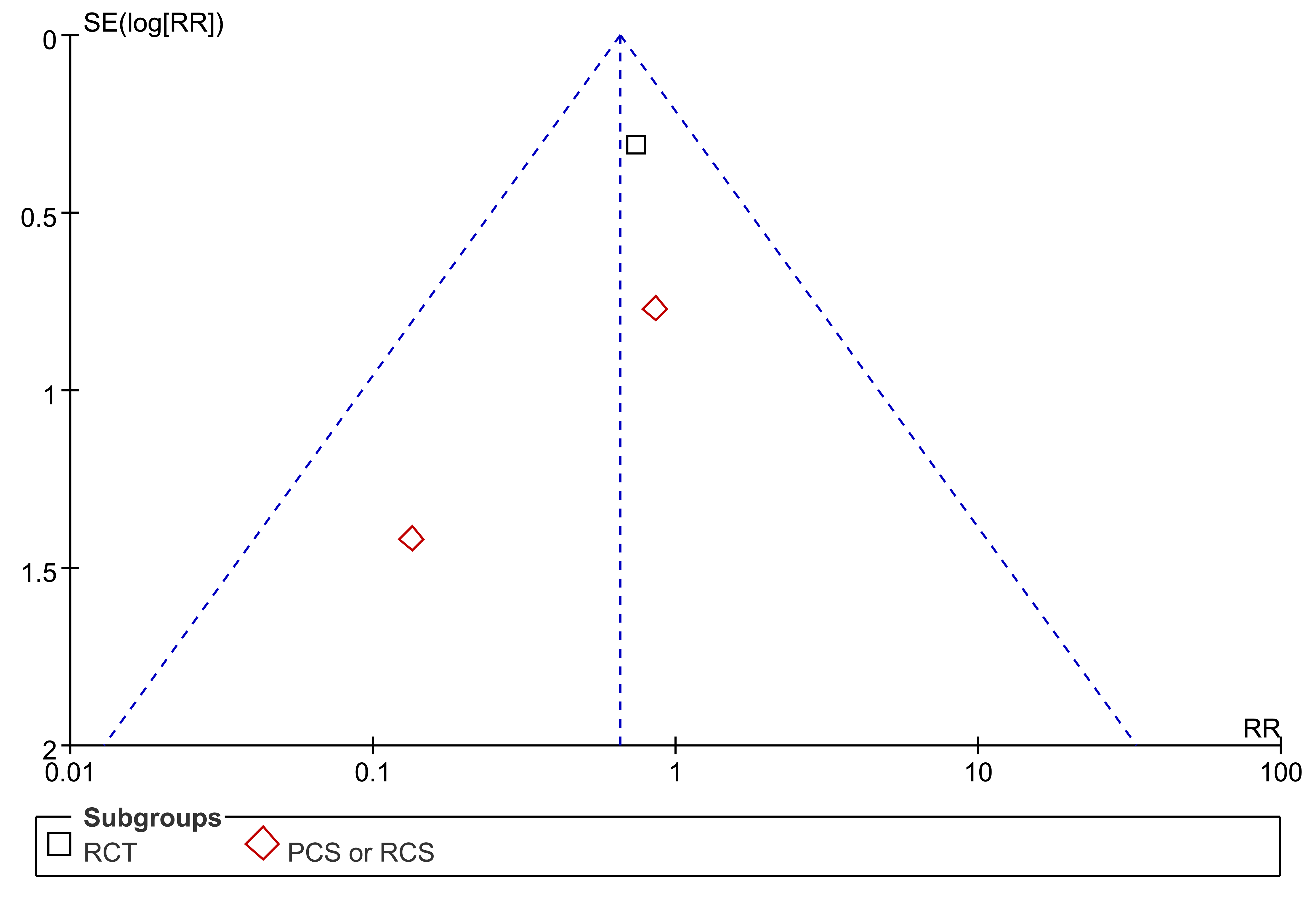


Figure S15 funnel plot of endocrine pancreatic insufficiency

**endoscopic step-up approach vs open necrosectomy**


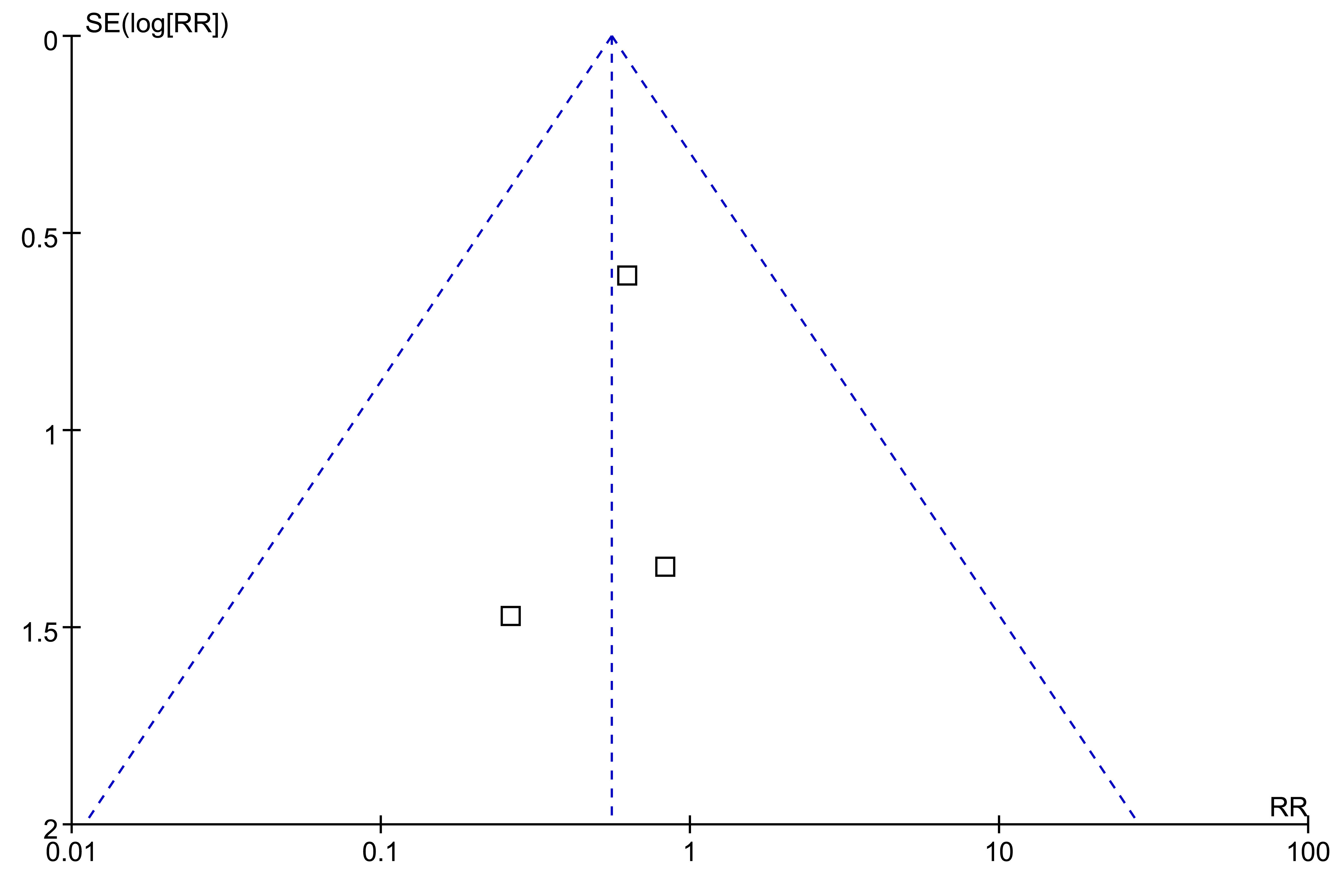


Figure S16 funnel plot of death


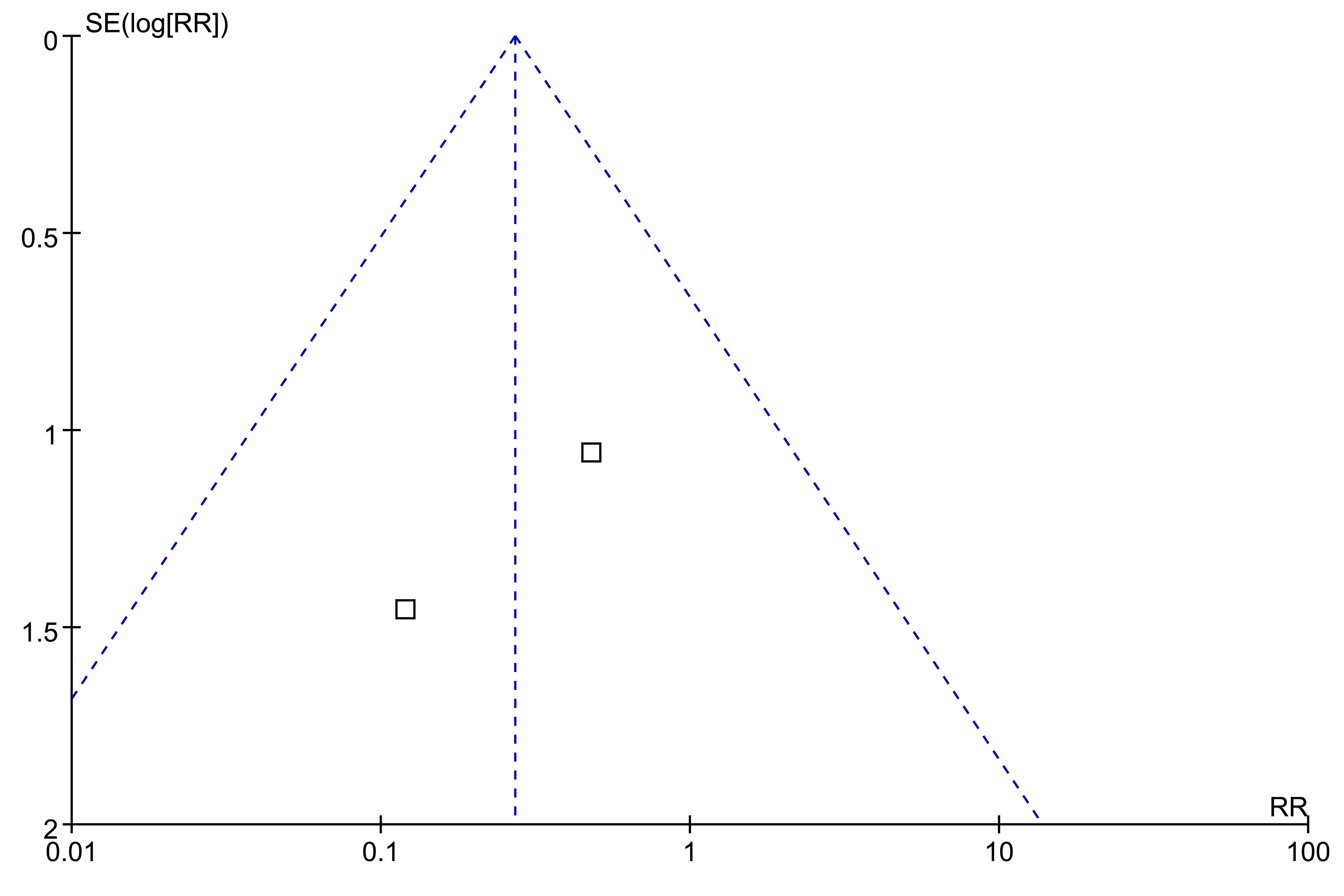
Figure S17 funnel plot of pancreatic-cutaneous fistula


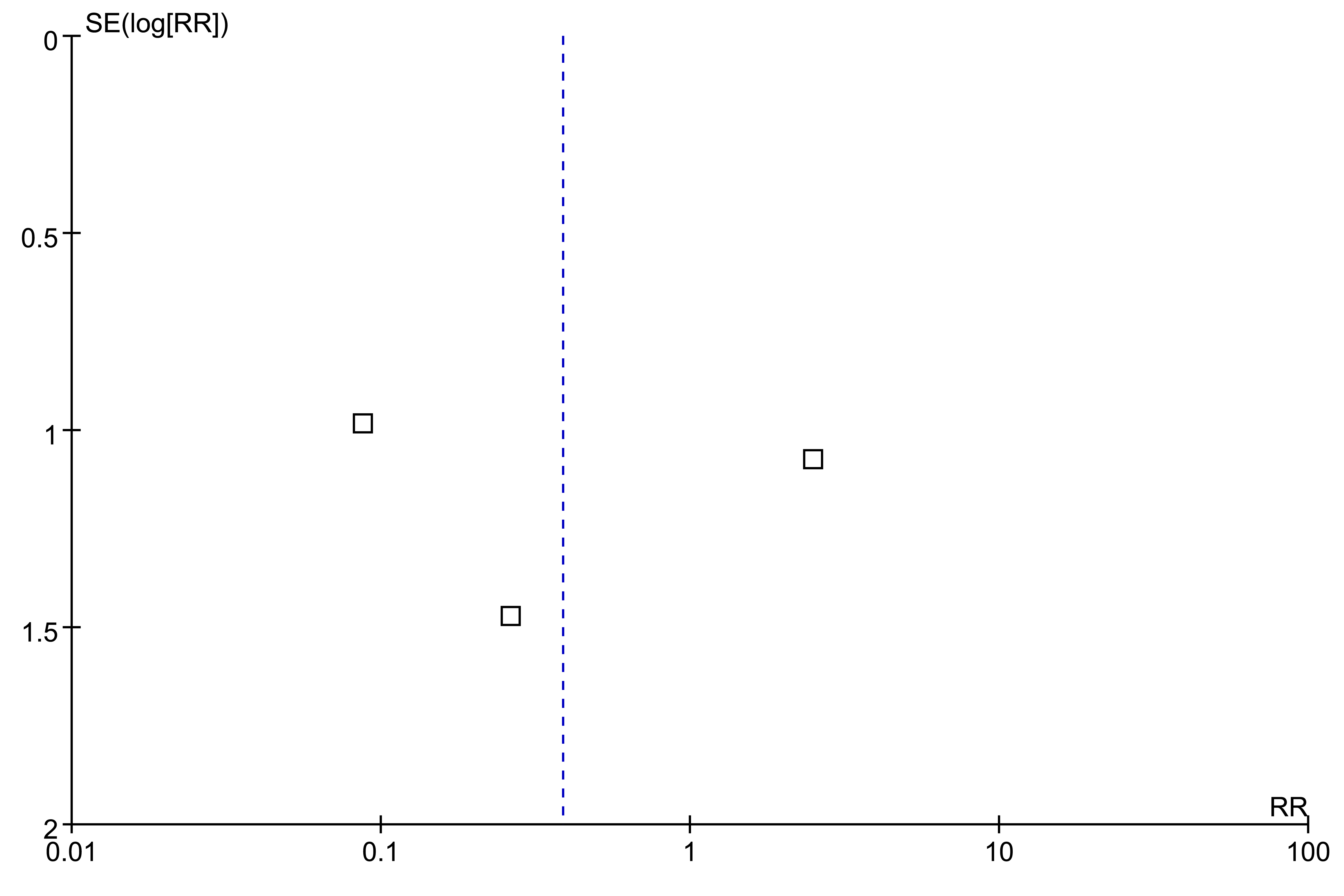


Figure S18 funnel plot of intra-abdominal bleeding


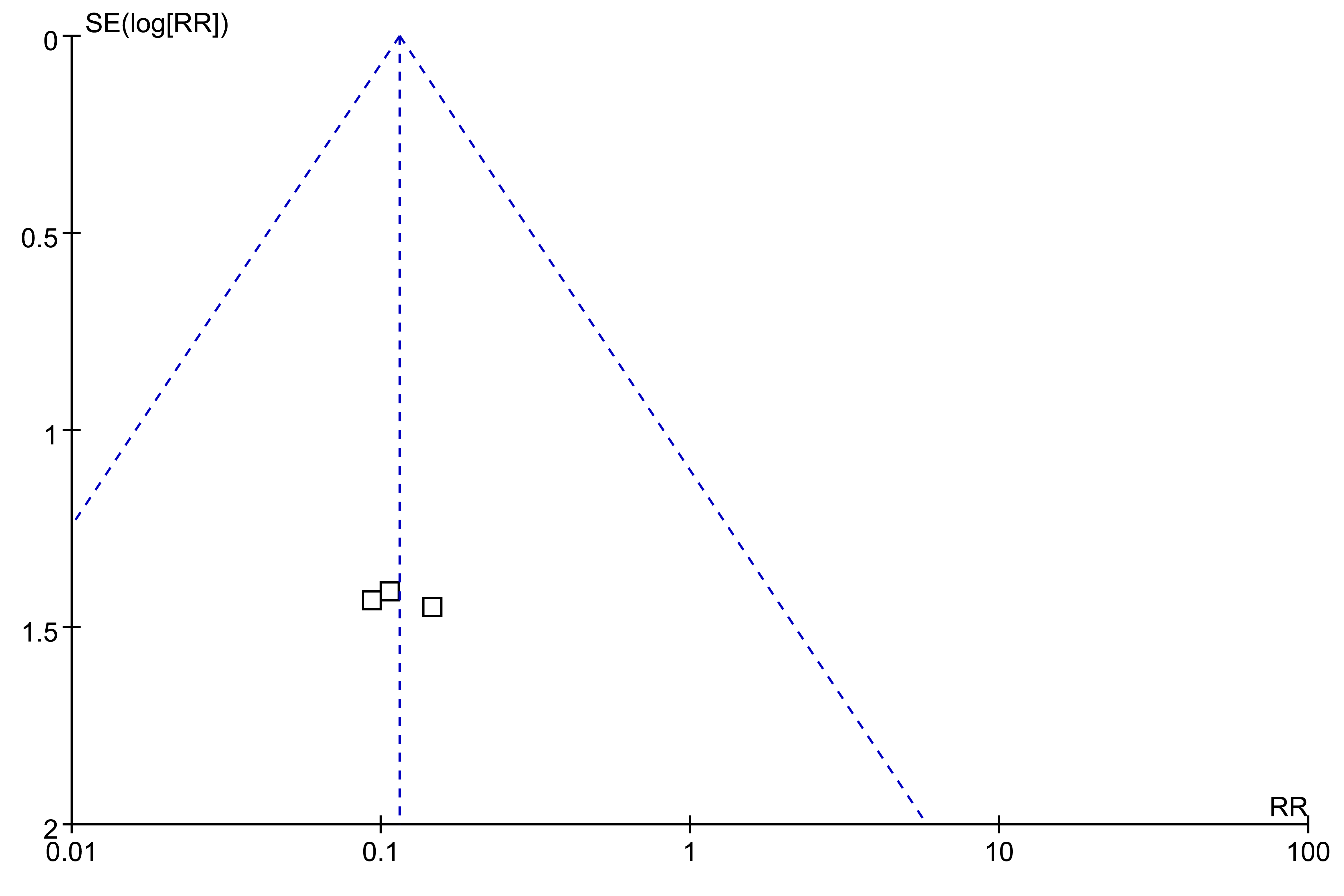


Figure S19 funnel plot of endocrine pancreatic insufficiency
